# GC–MS-based urinary organic acid profiling reveals multiple dysregulated metabolic pathways following experimental acute alcohol consumption

**DOI:** 10.1038/s41598-018-24128-1

**Published:** 2018-04-10

**Authors:** Cindy Irwin, Lodewyk J. Mienie, Ron A. Wevers, Shayne Mason, Johan A. Westerhuis, Mari van Reenen, Carolus J. Reinecke

**Affiliations:** 1 Centre for Human Metabolomics, Faculty of Natural Sciences and Agriculture, North-West University (Potchefstroom Campus), Private Bag, X6001 Potchefstroom, South Africa; 2 Radboud University Nijmegen Medical Centre, Translational Metabolic Laboratory, Department of Laboratory Medicine, PO Box 9101, 6500 HB Nijmegen, The Netherlands; 3 Biosystems Data Analysis, Swammerdam Institute for Life Sciences, University of Amsterdam, Amsterdam, The Netherlands; 4 Department of Statistics, Faculty of Natural Sciences and Agriculture, North-West University (Potchefstroom Campus), Private Bag, X6001 Potchefstroom, South Africa

## Abstract

Metabolomics studies of diseases associated with chronic alcohol consumption provide compelling evidence of several perturbed metabolic pathways. Moreover, the holistic approach of such studies gives insights into the pathophysiological risk factors associated with chronic alcohol-induced disability, morbidity and mortality. Here, we report on a GC–MS-based organic acid profiling study on acute alcohol consumption. Our investigation — involving 12 healthy, moderate-drinking young men — simulated a single binge drinking event, and indicated its metabolic consequences. We generated time-dependent data that predicted the metabolic pathophysiology of the alcohol intervention. Multivariate statistical modelling was applied to the longitudinal data of 120 biologically relevant organic acids, of which 13 provided statistical evidence of the alcohol effect. The known alcohol-induced increased NADH:NAD^+^ ratio in the cytosol of hepatocytes contributed to the global dysregulation of several metabolic reactions of glycolysis, ketogenesis, the Krebs cycle and gluconeogenesis. The significant presence of 2-hydroxyisobutyric acid supports the emerging paradigm that this compound is an important endogenous metabolite. Its metabolic origin remains elusive, but recent evidence indicated 2-hydroxyisobutyrylation as a novel regulatory modifier of histones. Metabolomics has thus opened an avenue for further research on the reprogramming of metabolic pathways and epigenetic networks in relation to the severe effects of alcohol consumption.

## Introduction

Notwithstanding the encyclopaedic information on alcoholism, the WHO asserts that alcohol remains one of the world’s leading risk factors for disability, morbidity and mortality — 5.9% of all deaths worldwide are attributable to alcohol consumption, exceeding those from HIV/AIDS (2.8%), violence (0.9%) or tuberculosis (1.7%)^1^. The tenth edition of the *International Classification of Diseases* lists at least 25 chronic conditions that are entirely attributable to alcohol; alcohol is also a risk factor in certain cancers, some tumours, numerous cardiovascular and digestive diseases, and many neuropsychiatric conditions^2^. Brain image studies have revealed changes in brain structure during the progression from adolescence to adulthood^3,4^, a critical period characterized by increased brain connectivity and maturation of brain neural circuits. These changes are highly susceptible to the effects of exogenous substances, which likely include alcohol^5–7^, making children and adolescents especially vulnerable to alcohol-related harm^8^. It was reported from a recent survey that approximately 50% of children (aged 11–14 years) in the UK have consumed alcohol, and 33% of adolescents (15–16 years) admitted to having experienced at least one episode of acute alcohol intoxication in the month preceding the survey^9^.

Human genome-wide association studies (GWAS) have identified polymorphisms and candidate genes associated with individuals’ innate risks for alcohol dependence^10–12^. With metabolomics involving large-scale molecular studies of metabolic systems, it provides novel and complementary approaches to GWAS studies^13–15^. Additionally, metabolomics has revealed the extensive perturbations of various metabolic pathways in response to chronic alcohol consumption, which underpin alcohol-induced disease states. Such studies show much promise for disease profiling and biomarker identification of the conditions associated with and arising from chronic alcohol consumption^16^. The burden attributable to acute alcohol consumption (rapid ingestion of alcoholic beverages) or binge drinking (drinking too much too fast) has been shown to be similarly high^17–19^. Impediments associated with acute and binge drinking include perturbed metabolism (e.g. glycogen depletion, acidosis and hypoglycaemia)^20^, damage to intestinal epithelial cells^21^, neurobiological diseases^22^, and ultimately even abrupt premature death due to physical or mental disabilities^23,24^.

Several important observations on acute alcohol consumption have recently been obtained from animal-based studies:

1. A rodent “intragastric feeding model” was investigated with ultra-high performance liquid chromatography-time of flight mass spectrometry (UHPLC-TOFMS) technology to determine changes in global metabolite profiles for plasma and urine from alcohol treated rats and mice compared to control animals^25^. Apart from several other observations, the researchers reported changes in the concentrations of 5-hydroxytryptophan and xanthurenic acid, both of which are intermediates in tryptophan metabolism. These observations provide further insight into the association of liver metabolism in response to ethanol exposure.
2. A study on the immune response of healthy individuals (11 males and 14 females, aged 21–56 years) with no history of alcohol use disorder, revealed that acute binge drinking resulted in a rapid increase in serum Gram-negative bacterial endotoxin lipopolysaccharide (LPS) and bacterial 16S rDNA. LPS is a potent trigger of the inflammatory cascade via activation of the Toll-like receptor 4 (TLR4) complex and increase in the portal and/or systemic circulation in several types of chronic liver diseases^26^. The elevation of bacterial 16 S rDNA levels after acute binge drinking indicated transient gut-derived microbial translocation as a likely mechanism for the serum LPS increase. The authors suggest that the increased serum levels of bacterial products following acute consumption might contribute to innate immune responses and potentially to the behavioural effects associated with alcohol binge drinking. The study also draws attention to potential perturbations in metabolite profiles which may be due to the role of the gut and the microbiome in binge drinking. This point is substantiated by GC–MS urinary metabolomics results from another study, which revealed metabolite differences between Sprague–Dawley and Wistar rats following different perturbations, including consecutive acute ethanol interventions^27^. These results directed to different metabolic pathways and differences in the intrinsic metabolism and symbiotic gut microflora between these animal strains.
3. A review of the analytical technologies used in profiling studies of animal or human serum, plasma, urine and tissue samples, obtained following exposure to alcohol, summarizes a range of endogenous metabolites that have been proposed as potential ethanol consumption-related biomarkers^28^. This range of biomarkers provides biochemical insights that are essential for understanding the effects and mechanisms of ethanol toxicity.

Notwithstanding the potential of metabolic profiling, a recent review noted that few studies on acute alcohol consumption have been undertaken in humans^29^, recognizing that these models could provide a basis for studying the biochemical effects of prolonged ethanol exposure, as well as to potentially identify biomarkers for monitoring the progression of alcoholism in man^25^.

We thus postulated that our metabolomics approach could provide further insights into the metabolic signature which arises from a single excessive dose of alcohol. However, the response to experimental alcohol consumption is complex, as shown by the diverse observations noted from the study on two related animal strains^27^. Alcohol studies in humans, likewise, vary greatly according to the extent and method of usage (chronic or acute), individual variability (genetic and behavioural), and environmental factors. Also, individuals’ attitude to their consumption habits is a private issue, and important ethical considerations (such as restricted case participation and policy guidelines) have to be taken into account during the selection of sufficient and appropriate participants for the laboratory assessment of alcohol use^30^. Therefore, recording the influence of alcohol in laboratory studies involving a select group of moderate-drinking young men, as we present here, is complex from several points of view.

Despite these limitations and qualifications, the application of metabolomics to intervention or challenge studies is a preferred practical approach towards a holistic understanding of the effects of consumed substances on metabolism^31,32^. Intervention studies produce extensive data sets due to longitudinal (time-dependent), multi-subject (several experimental participants), multi-group (number of interventions), and multivariate data (numerous metabolic variables) inputs^33,34^. Our previous intervention study on consuming commercial flavoured water with a benzoic acid preservative^35^ indicated that time-dependent metabolomics investigations, using designed interventions, provide a way of interpreting the variation induced by the different factors of a designed experiment. This approach has the potential to significantly further our understanding of normal and pathophysiological perturbations of endogenous or exogenous origin. Here we report on a metabolomics study which used commercial flavoured water as a vehicle^35^ for vodka consumption, to simulate acute but controlled alcohol consumption (resembling a single incident of acute or binge drinking) in young male participants. All metabolomics studies are inherently hampered by an analytical inadequacy to provide a comprehensive coverage of the metabolome. Given the known main pathways of alcohol metabolism, we selected a gas chromatographic–mass spectrometric (GC–MS) approach to analyse the urinary samples collected during this time-dependent cross-over study. These analyses generated an extensive data set, from which (by using conventional and extended statistical methods) we could reveal a multitude of disturbances in the urinary organic acid profiles over time due to the alcohol intervention.

## Results

### Effect of the acute alcohol intervention across all cases

**Figure 1:**
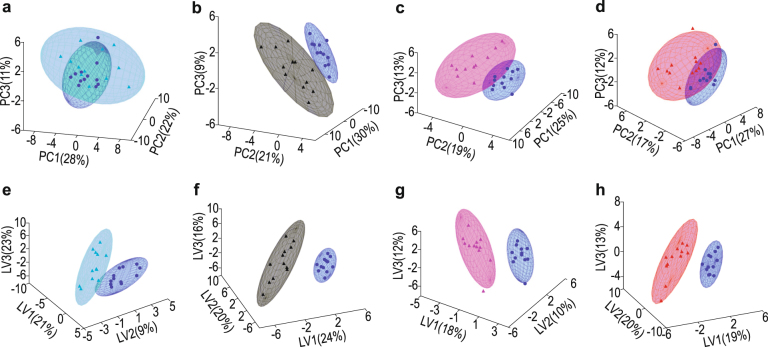
PCA and PLS–DA scores plots following alcohol consumption. Input data were from the 120 quantified metabolites for time 0 vs time 1 (**a**,**e**), time 0 vs time 2 (**b**,**f**), time 0 vs time 3 (**c**,**g**), and time 0 vs time 4 (**d**,**h**) following alcohol consumption. The samples for time 0 were collected just prior to the intervention, and are therefore regarded as the control samples. The figures were based on the non-paired method of analysis. Results from the paired method are shown in Supplementary Fig. S2.

Observations of the behaviour of the participants during the intervention stage indicated a moderate degree of acute alcohol consumption characteristic of the early phase of a binge-drinking event by young male students^36^. To determine the effect of the alcohol intervention, the data sets of 120 organic acid metabolites, generated from the samples collected at times 1, 2, 3 and 4 hours following the intervention, were compared with the data set from the samples collected prior to the intervention (time 0), and presented for unsupervised principal component analysis (PCA) (Fig. 1a–d). Subsequently, a supervised partial least squares discriminant analysis (PLS–DA) was performed to maximize the discrimination between the controls (time 0) and the subsequent hourly-collected data. The PCA scores plots showed some differentiation 1 hour after the alcohol consumption (Fig. 1a), followed by complete separation after 2 hours, and a progressive return to the time 0 profile after 3 and 4 hours. The PLS–DA scores plots (Fig. 1e–h) showed a complete separation for all four times following alcohol consumption relative to time 0.

**Figure 2:**
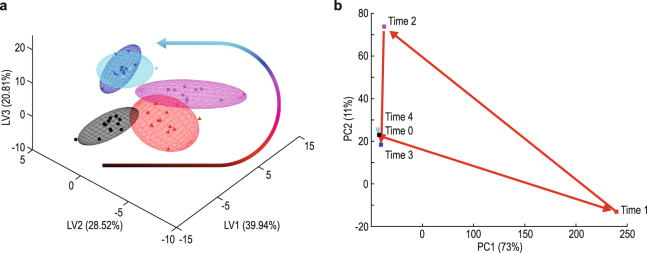
Multivariate approaches to indicate the time effect. (**a**) ASCA scores on the first three latent variables (LV1 to LV3), along with 95% confidence ellipsoids for the centroids, are shown and colour-coded according to time following the intervention (0, 1, 2, 3 and 4 hours shown in black, red, pink, dark blue and light blue, respectively), with the arrow showing the time-dependent trend, using the same discriminating colour sequence. (**b**) Unfolded PCA scores centroids for each time point based on the first two principle components (PC1 and PC2) are shown. The colours of the centroids are the same as for the ASCA and the direction of the trajectory is indicated by the red arrows, starting from time 0.

For better insight into the time effect of the intervention, two complementary approaches were used, both of which are founded on Principal Component Analysis (PCA). The first, ANOVA Simultaneous Component Analysis (or ASCA), enabled us to partition variation similar to an analysis of variance (or ANOVA) approach using the experimental design, and summarize variation observed across many variables through projection to a lower dimensional space. The scores plot resulting from the ASCA model (Fig. 2a) indicates a marked change one hour after the intervention. After 3 hours a state of homeostasis is reached as indicated by the similarity between times 3 and 4. This is, however, a new state of homeostasis, since it is not comparable to the state prior to the intervention, as will be discussed below. Though time 2 appears to show a larger perturbation compared to time 1, following the intervention, time 2 also shows a progression towards the new state of homeostasis, making it less indicative of the initial alcohol effect. The ASCA model partitioned variation from various sources in the design (that is, time and participant) by averaging out individual effects. Secondly, performing PCA on the dataset unfolded in time provides another view on the variation observed, which ties repeated observations together into a single profile. The scores from such a model provide a complimentary and, in a sense, confirmatory summary of the effect of the alcohol consumption in time, as indicated by the score centroids shown in Fig. 2b. It is evident that there was a change in the organic acid profile (i.e. across all cases) over time, with the most extensive change visible 1 hour (time 1) after the intervention. The centroids plotted for times 2 to 4 complement the interpretation made from the ASCA.

Based on the statistical criteria, a total of 13 variables (Table 1) were identified as those responsible for the separation between the control samples and those collected one hour after alcohol consumption. Shown in Table 1 are the VIP values, *p*-values and FC values at time 1 relative to time 0, as well as the mean and standard deviation values for all times. The FC and *p-*values for time 4 relative to time 0 are also included to show the eventual diminishing effect of the alcohol on the 13 metabolites. In addition, since ethanol data is essential in the context of this intervention study, these values are included for reference purposes. However, ethanol cannot be reliably detected with the GC–MS method used here, and therefore the quoted urinary ethanol values were measured through a targeted proton nuclear magnetic resonance [^1^H-NMR] spectroscopy analysis. The full metabolite profile that became apparent through this NMR metabolomics study will be reported in a subsequent paper.

**Table 1:** Univariate, multivariate and descriptive statistics for the most perturbed metabolites following alcohol consumption.

Metabolites^a^	PLS**–**DA VIP ≥ 1.0 (Controls vs 1 hr)	[WRT]_0−1_	[FC]_0−1_	Mean concentrations of metabolites [Std. Dev.] μmol metabolite/mmol Cr					[WRT]_0−4_	[FC]_0−4_	HMDB Ref. values μmol metabolite/mmol Cr
		Summary statistics (Controls vs 1 hr)		t = 0	t = 1	t = 2	t = 3	t = 4 hr	Summary statistics (Controls vs 4 hr)		
Hippuric acid	19.6	0.002	+3.2	948 [737]	3037 [1357]	2775 [2051]	1157 [1111]	726 [764]	0.239	−1.3	27.9–932.7
Lactic acid	14.4	0.015	+27.4	8.73 [3.65]	239 [412]	28.1 [19.7]	15.5 [14.7]	12.1 [8.51]	0.209	+1.4	3.9–9.8
Fumaric acid	7.24	0.003	+6.4	0.78 [0.47]	4.96 [4.53]	5.88 [6.08]	2.62 [1.94]	2.8 [2.15]	0.006	+3.6	0.75–1.2
Vanillylmandelic acid	5.03	0.034	−1.6	15.4 [3.61]	9.87 [7.39]	2.96 [2.32]	1.87 [1.11]	1.75 [0.99]	0.002	–8.8	1.1–1.7
2-Hydroxybutyric acid	4.06	0.034	+4.3	2.68 [1.54]	11.4 [16.0]	3.85 [2.68]	2.26 [1.16]	2.52 [2.15]	0.695	−1.1	1.2–6.9
Succinic acid	3.88	0.010	+2.6	3.81 [3.81]	9.83 [7.90]	20.2 [24.6]	12.8 [12.8]	12.7 [12.3]	0.004	+3.3	4.9–14.9
3-Hydroxybutyric acid	2.76	0.041	+5.9	0.40 [0.54]	2.39 [2.96]	0.59 [0.55]	0.47 [0.40]	0.77 [0.68]	0.182	+1.9	1.4–2.2
2-Ethylhydracrylic acid	1.66	0.010	+2.1	1.09 [1.00]	2.30 [1.73]	2.53 [2.78]	1.76 [1.61]	1.86 [1.47]	0.015	+1.7	1.3–2.9
3-Hydroxyisobutyric acid	1.56	0.034	+1.9	3.01 [1.27]	5.76 [3.86]	3.31 [2.05]	2.74 [1.25]	3.44 [1.95]	0.239	+1.1	4.1–19.0
2-Hydroxyisobutyric acid	1.39	0.034	+1.8	11.1 [4.03]	20.5 [14.2]	20.5 [13.4]	12.8 [6.93]	16.3 [13.1]	0.209	+1.5	4.4–7.6
Malic acid	1.32	0.023	+4.9	0.27 [0.16]	1.30 [1.60]	1.81 [1.59]	0.86 [0.67]	1.19 [1.37]	0.006	+4.5	0.7–5.3
N-Tiglylglycine	1.22	0.034	+1.7	1.55 [1.15]	2.65 [1.39]	10.9 [9.15]	10.9 [9.35]	10.8 [8.64]	0.002	+7.0	0.78–1.2
2-Hydroxyglutaric acid	1.06	0.023	+1.7	2.88 [0.99]	4.91 [3.39]	10.0 [5.90]	7.22 [3.62]	8.50 [5.73]	0.003	+3.0	0.8–52.0
Ethanol^b^	n/a	n/a	n/a	0.0	1368	5173	2852	1810	n/a	n/a	n/a

A total of 13 organic acid metabolites were identified as important discriminatory variables one hour after alcohol consumption (VIP ≥ 1.0, *p* ≤ 0.05 and |FC| ≥ 1.5). Abbreviations used: [Std. Dev.], standard deviation; Cr, urinary creatinine value; HMDB, Human Metabolome Database; [WRT]_0–1_ and [WRT]_0–4_, Wilcoxon signed-rank test *p*-values showing the significance of a metabolite at times 1 and 4 hours relative to time 0; [FC]_0–1_ and [FC]_0–4_, fold change values for a metabolite at times 1 and 4 hours relative to time 0; n/a, not applicable for the purpose of this table.

^a^13 organic acid metabolites (concentrations expressed as μmol/mmol Cr), identified through GC–MS analysis.

^b^Ethanol (concentrations expressed as μmol/mmol Cr), identified through an independent, targeted ^1^H-NMR analysis of the same urine samples as used for the GC–MS analyses.

Consumed ethanol is known to be dispersed through exhaling (not measured), metabolic conversions and urinary excretion, which peaked at two hours following its consumption (Table 1). However, the results from the ASCA and unfolded PCA (Fig. 2) — which indicated that the most dramatic metabolite changes occurred one hour following the intervention — encouraged us to preferably study in detail the metabolic effects seen one hour after alcohol consumption. Of the important metabolites, hippuric acid, the phase II biotransformation product of benzoic acid^27,35–38^, had the highest VIP value (Table 1, VIP = 19.6). The concentration of hippuric acid was already high at time 0 (948 μmol/mmol Cr), due to its being the normal excretion product of benzoic acid derived from the gut microbiome. This concentration increased to 3037 μmol/mmol Cr one hour after the alcohol consumption, owing to the benzoic acid preservative in the flavoured water vehicle consumed with the alcohol. Hippuric acid likewise appeared to be the most important discriminating variable in the previously described effects of the vehicle-only intervention^35^, as well as in the consecutive acute ethanol intervention study on rats, where the change in urinary hippuric acid is suggested to be due to a metabolic dysfunction of damaged liver tissue^27^. The presence of the high concentration of hippuric acid does not influence the outcome of the analysis, since the same list of important metabolites (VIP > 1.0) is obtained after excluding hippuric acid from the data (see section 4.6 of the SI). It is also worth noting that four other gut-related urinary metabolites observed in the rat study^27^ were also present in the urine samples from our study (see Supplementary Table S2 in section 3 of the SI), but did not appear to be discriminatory metabolites due to the alcohol intervention. Other important biotransformation products from the rat and mouse intervention studies, such as ethyl glucuronide and ethyl sulphate^25^, were not detectable by the GC–MS methods used in the present study.

The metabolic interrelations among the remaining 12 metabolites in Table 1 correspond to several metabolic consequences of alcohol consumption. The oxidation of ethanol by alcohol dehydrogenase (ADH) creates a highly reduced cytosolic environment in hepatocytes, and favours the production of lactic acid (second-highest VIP) from pyruvic acid, resulting in downstream metabolic consequences due to pyruvic acid depletion. The reduced environment also accounts for the perturbations observed for vanillylmandelic (FC = −1.6), 2-hydroxybutyric (FC = +4.3) and 3-hydroxybutyric (FC = +5.9) acids, and increased urinary excretion of succinic, fumaric, malic and 2-hydroxyglutaric (derived from 2-ketoglutaric acid) acids that implicates a dysfunctional Krebs cycle. Increased excretion of N-tiglylglycine (the phase II biotransformation product of tiglyl-CoA) and 2-ethylhydracrylic acid (intermediates in the R- and S-pathways of isoleucine catabolism, respectively), as well as 3-hydroxyisobutyric acid (produced from valine catabolism) points to amino acid mobilization but inhibition of gluconeogenesis^39^. The reason for the increased excretion of 2-hydroxyisobutyric acid (2-HIBA) remains speculative and will be discussed below.

**Figure 3:**
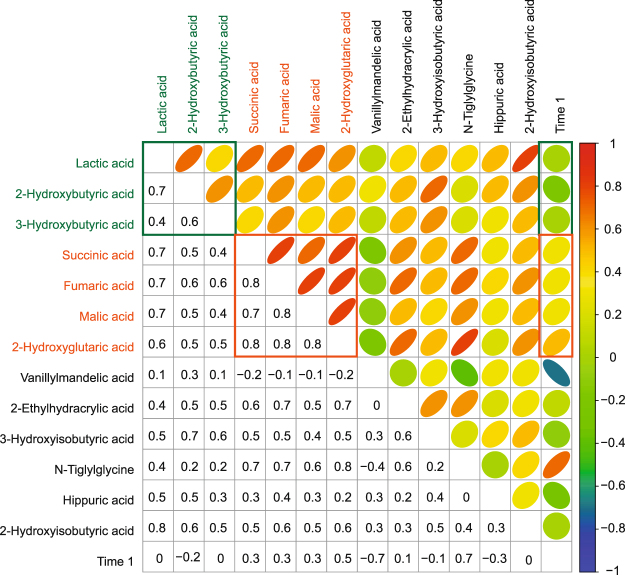
Correlation matrix over the full time period for the 13 metabolites listed in Table 1. Correlations extend from high positive (red; r ≥ 0.6) through neutral (green; −0.2 < r < 0.2) to high negative (blue; r ≤ −0.6). The high correlation between lactic, 2-hydroxybutyric and 3-hydroxybutyric acids and the high correlation between the Krebs cycle-associated metabolites (together with their respective time slots) are blocked in green and orange, respectively.

To characterize the metabolic relationship between the 13 important variables further, we calculated Spearman’s rho correlation coefficients over the full period of the alcohol intervention, as described in section 4.4 of the Supplementary Information (SI), and shown in Fig. 3.

Important observations from the correlation analysis correspond to the metabolic interrelations: (1) the profile for vanillylmandelic acid was unique — it was the only metabolite that showed a negative correlation with time (r = −0.7), indicating that its urinary concentration constantly decreased over the study period, and remained very low (1.75 μmol/mmol Cr; *p = *0.034; FC = −8.8) towards the end (at time 4 hours). (2) A good correlation (r = 0.4 to 0.7) was observed between the indicators of ketosis (lactic, 2-hydroxybutyric and 3-hydroxybutyric acids), but only in the initial phase following alcohol consumption, as indicated by their neutral correlations with time (r = 0 to −0.2 with time). (3) A high to very high correlation (r = 0.6 to 0.8) was observed between the Krebs cycle-associated metabolites, all of which also show a reasonable correlation (r = 0.3 to 0.5) over the full period following the intervention. (4) Likewise, N-tiglylglycine showed a high correlation with the Krebs cycle intermediates (r = 0.6 to 0.8), which also extended over the full study period (r = 0.7).

The high correlation values shown in Fig. 3 collectively substantiate a strong interrelationship between the 13 organic acid metabolites that were regarded as important due to acute alcohol consumption. The mean concentrations, and the time 4 relative to time 0 *p*-values ([WRT]_0–4_) and fold changes ([FC]_0–4_) for the 13 variables (listed in Table 1), support the conclusions about the individual and group correlations observed in Fig. 3; they also provide an alternative and additional support for the observations from the correlation study.

### Inter-individual variation following acute alcohol consumption

In order to illustrate the inter-individual variation between the participants, as well as the longitudinal effect of the alcohol consumption, PCA of the data unfolded in time was performed as described in section 4.5 of the SI. The unfolding transformed the three-dimensional data (a tensor of cases, interventions and time) into a two-dimensional matrix, and thus allowed PCA to account for the longitudinally repeated measures.

**Figure 4:**
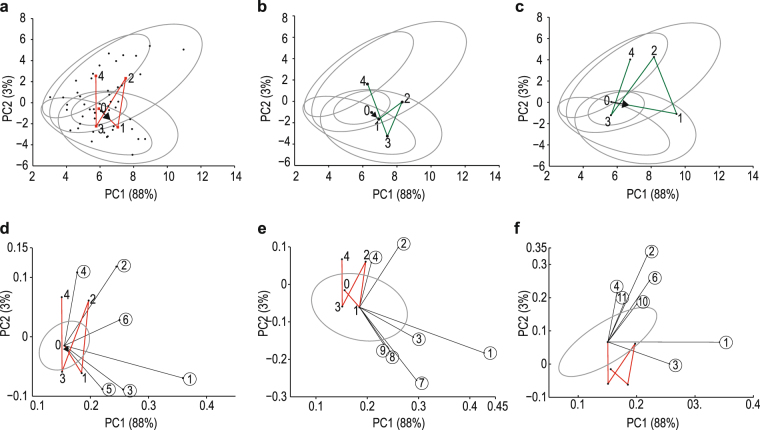
Unfolded PCA scores and selected bi-plots. (**a**) PC1 and PC2 of the mean of the cases studied are shown (in red), as well as the 90% confidence ellipsoids for scores of PC1 and PC2 at time 0, 1, 2, 3 and 4 hours. The centroids of the five time-dependent clusters are indicated as red squares. The direction of the trajectory linking the centroids from time 0 onwards is indicated by a black arrow. (**b**) and (**c**) The trajectories of the spectral profiles of two individual cases, illustrating the variation in the averaged profiles in response to the alcohol intervention. (**d**–**f**) Bi-plots showing the seven most important metabolites when ranking was based on the sum of the squares of loadings of the first two components. These metabolites are more primarily responsible for the pattern in the plot, that is, causing separation in the unfolding of the PCAs, as applicable for times 0 (**d**), 1 hour (**e**) and 4 hours (**f**). Encircled numbers identify the metabolites: 1, hippuric acid; 2, phenylacetylglutamine; 3, 4-hydroxyphenylacetic acid; 4, indole-3-acetic acid; 5, pyrrole-2-carboxylic acid; 6, pyroglutamic acid; 7, lactic acid; 8, 2-hydroxybutyric acid; 9, fumaric acid; 10, N-cinnamoylglycine; 11, glucuronic acid.

Figure 4a–c illustrates the scores based on the first two principal components (PC1 and PC2) of the PCA model. This analysis of the unfolded data provides insight into the effect of the acute alcohol consumption over time on the 120 metabolites. The centroids of the PCA scores for each time (Fig. 4a), as well as those of two individual cases (Fig. 4b,c), illustrate the inter-individual variation in response to the intervention. The averaged and individual trajectories showed similarities in the individual responses to the alcohol consumption over time, since they all indicated a biphasic response pattern (phase 1: times 0 to 1 to 2, and phase 2: times 2 to 3 to 4). However, distinct differences were also noted, indicated by the unique orientation and biphasic profiles of the trajectories of the individuals (Fig. 4b,c). The metabolic basis for the latter observation is illustrated, for example, by a comparison of the lactic acid excretion observed in the two cases, which progressed from 8.2 to 6.0, 74.5, 14.7 and 16.0 μmol/mmol Cr for case 1 (Fig. 4b), but from 10.6 to 31.2, 19.8, 8.7 and 11.2 μmol/mmol Cr for case 2 (Fig. 4c). Similar differences in the individual responses to the alcohol consumption were observed for several other metabolites as well, which cumulatively result in the unique trajectory of each individual subject — an observation that agrees with the contemporary view on genetic and metabolic individuality.

Figure 4d–f illustrates the bi-plots for the top most influential metabolites responsible for the separation in the unfolding of the PCAs. The dominant metabolites observed at time 0 were phase II biotransformation products (hippuric acid and phenylacetylglutamine) and organic acids originating from the gut microbiome (4-hydroxyphenylacetic, indole-3-acetic, pyrrole-2-carboxylic and pyroglutamic acids). This gut-derived organic acid profile dominates at all the times measured, as also indicated in Figs. 4e,f for times 1 and 4 hours. Notably, one hour after alcohol consumption, lactic, 2-hydroxybutyric and fumaric acids appear as additional important metabolites, in accordance with the results in Table 1 and Fig. 3. At time 3, aconitic acid is indicated as an important metabolite (figure not shown), whereas N-cinnamoylglycine and glucoronic acid (both associated with phase II biotransformation) appear to be important 4 hours after alcohol consumption. It thus appears that the endogenous detoxification mechanisms through biotransformation remained functional despite the acute alcohol consumption.

## Discussion

**Figure 5:**
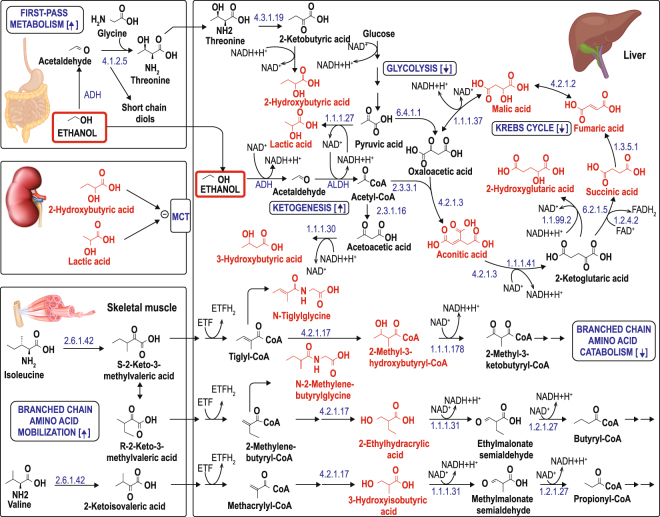
Proposed model indicating some important metabolic pathways affected by acute alcohol consumption. Up- or down-regulated metabolites are shown in red. Abbreviations used: MCT, monocarboxylic acid transporter; four-figure numbers (e.g. 2.6.1.42) refer to the IUBMB enzyme nomenclature (EC number); S, main catabolic pathway of isoleucine; R, minor catabolic pathway of isoleucine, which may act as a safety valve for overflow of accumulating metabolites from the S-pathway. Credit for images used: Kidney – ID 20446327 © Natis76 | Dreamstime.com; Muscle – ID 69693231 © Tigatelu | Dreamstime.com; Liver & Digestive system – ID 43552467 © Bluezace | Dreamstime.com.

In this study, the metabolomics organic acid profile revealed significant metabolic effects of a single dose of alcohol, consumed in a well-defined vehicle by healthy, moderate-drinking males. Although their analytical constraints limit the scope of profiling studies, we concur with the view that carefully conducted studies in humans are warranted, and would provide valuable new insights into the short and long term effects of alcohol exposure, alcoholic liver disease and alcoholism, in man^28^. The method followed here offered a convenient and sensitive approach to uncover perturbed metabolic pathways, of which we modelled the main ones as illustrated in Fig. 5. Several distinct insights deserve special attention.

The first insight deals with hepatic robustness. Glycine N-acyltransferase (GLYAT)-based biotransformation was shown to play a vital background role during the intervention study. The scores and bi-plots from the unfolded PCAs (Fig. 4d–f) revealed the reasons for the observed differentiation between the groups shown in the scores plots of the unsupervised PCAs (Fig. 1a–d). The observed differentiation was caused primarily by detoxification (gut metabolites) and biotransformation (phase II conjugates) processes. Apart from the alcohol load from the intervention, the liver is simultaneously confronted with the benzoic acid load from the vehicle, which induced a significant hepatic biotransformation response^35^. Two closely related acyl-CoA:amino acid N-acyltransferases have been characterized as operative in liver mitochondria^40^. One transferase was specific for benzoyl-CoA, salicyl-CoA, and certain short straight- and branched-chain fatty acyl-CoA esters as substrates, whereas the other enzyme specifically used either phenylacetyl-CoA or indoleacetyl-CoA. It thus seems probable that the benzoyl-CoA:glycine N-acyltransferase (using glycine as the preferred acyl acceptor) was functional in the biotransformation of the vehicle-induced accumulated benzoyl-CoA, but notably also of tiglyl-CoA and 2-methylenebutyryl-CoA, which accumulated from perturbations due to the alcohol consumption. This biotransformation resulted in the formation and increased excretion of hippuric acid, N-tiglylglycine and N2-methylenebutyrylglycine (a minor metabolite increased due to the alcohol consumption — data not shown, but included in Fig. 5), respectively. The metabolomics approach thus provides insight into a background process that underscores the robustness of hepatic function in healthy individuals, notwithstanding the impact of the acute alcohol consumption — an observation that would not hold true for the liver affected by chronic alcoholism.

The second insight deals with the comprehensive metabolic perturbations caused by the acute alcohol consumption, as disclosed by the supervised PLS–DA. The metabolic reactions following alcohol consumption showed a phased response profile, to which the gut is known to contribute following alcohol consumption. In the early phase, a fraction of the alcohol consumed orally may be immediately metabolized by the gut alcohol dehydrogenase (ADH) isoforms in a process known as first-pass metabolism^41^. A study on acute alcohol binge drinking showed increased serum levels of bacterial products (endotoxin and bacterial 16S rDNA) derived from the gut microbiome^42^. The serum endotoxin levels rapidly increased within 30 minutes following consumption, remained elevated for 3 hours and returned to lower than baseline levels by 24 hours after alcohol intake. The early increase in 2-hydroxybutyric acid following alcohol consumption observed in the present study (Table 1, Figs 3 and 4e metabolite 8) is an indication of a limited contribution from first-pass metabolism in the present experimental group: threonine is anticipated to be produced from the increased intestinal acetaldehyde (the product of ethanol metabolism), and is subsequently catabolised in the liver to 2-hydroxybutyric acid, as proposed in the representation shown in Fig. 5. However, since the liver is important in alcohol metabolism, and considering its wide spectrum of lesions^43^, we linked most of the metabolomic perturbations observed in the present study to the liver metabolism, as shown in the conceptual model in Fig. 5. Alcohol oxidation by hepatic ADH results in the reduction of NAD^+^ to NADH, thereby generating a highly reduced cytosolic environment in hepatocytes. The increased NADH:NAD^+^ ratio influences the acetaldehyde–lactate dehydrogenase coupled lactic acid accumulation (ALDH–LDH = EC1.2.1.10–EC1.1.1.27). Greatly increased lactic acid excretion (Table 1, Figs 3 and 4e metabolite 7) is proposed as the next early hallmark of acute alcohol consumption and, together with the concomitant early increase in 3-hydroxybutyric acid (Table 1 and Fig. 3), is linked to ketogenesis. Acetoacetic acid, the precursor of 3-hydroxybutyric acid, showed a similar early increase (0.012, 0.016 and 0.044 μmol/mmol Cr at times 0, 1 and 2 hours, respectively), and peaked at two hours after alcohol consumption (VIP = 0.004; FC = 3.64; *p* = 0.02).Taken together, first-pass metabolism and ketogenesis seem to be the immediate salvage responses to disturbed homeostasis caused by the acute alcohol consumption.

Further, an increased NADH:NAD^+^ ratio (depleted NAD^+^) has been described to influence the direction of several metabolic processes, including decreased glycolysis^44^, decreased Krebs cycle^20,45^, and decreased gluconeogenesis^20,46^. The early increase in fumaric acid (Table 1, Figs 3 and 4e metabolite 9) indicates reduced Krebs cycle functionality, which persisted to the end of the 4-hour period of the present intervention study (Table 1 and Fig. 3). The notable and persistently reduced Krebs cycle functionality is further supported by the inhibition of the catabolism of all three branched-chain amino acids, indicated by the profiles of N-tiglylglycine, 2-ethylhydracrylic acid and 3-hydroxyisobutyric acid (Table 1 and Fig. 3). It is worth noting that the perturbed urinary metabolite profile had not normalized 4 hours after alcohol consumption, although the supervising physician regarded the participants sufficiently sober to permit them to leave the clinic at this time.

The third insight highlights the observations on vanillylmandelic acid. This metabolite was significantly down-regulated by depleted NAD^+^, and remained so over the full 4-hour period (Table 1 and Fig. 3). The down-regulation of vanillylmandelic acid reflects the upstream inhibition in the catabolism of the catecholamines noradrenaline and adrenaline, which occurs due to the inhibition of the NAD^+^-dependent aldehyde dehydrogenase-catalysed reactions (EC1.2.1.3 and EC1.2.1.5, respectively). The known relative rise in adrenaline and the delayed increase in noradrenaline concentrations after alcohol consumption is a physiological effect of the inhibition of these enzymes^47^, which, together with the corticotrophin-releasing factor, may contribute to negative affective states and relapse vulnerability during alcohol abstinence^48^. The observations on vanillylmandelic acid may also be important for other alcohol related studies. In a study on alcoholic patients, higher levels of urinary homovanillic acid (an up-stream metabolite of the catecholamine pathway) were observed in patients with a A1 allele in their genotype, when compared to patients homozygous for the *Taq*IA2 allele^49^. We observed that homovanillic acid peaked two hours after acute alcohol consumption (VIP = 0.129; FC = 1.13; *p* 0.44). These findings suggest that determination of urinary levels of catecholamine end-products should be included in alcohol-related studies, since variance in their levels could relate to a polymorphism, and could affect addictive behaviour.

As a last insight, 2-hydroxyisobutyric acid (2-HIBA) was indicated in this study to be an important and significant organic acid indicator regarding the metabolic consequences of acute alcohol consumption. 2-Hydroxybutyric acid and 2-HIBA are normally reabsorbed in the kidney by a monocarboxylic acid transporter (MCT). This transporter is, however, also responsible for transporting lactic acid between the urine and the blood. After alcohol consumption, the increased lactic acid contributes to the saturation of these transporters. Consequently, not all of the 2-hydroxybutyric acid and 2-HIBA is reabsorbed, leading to their increased excretion in the urine. However, the conventional view is that urinary 2-HIBA is a non-metabolite, obtained from environmental exposure to tertiary-butylacetate (a commercial solvent used in industrial coatings and cleaners) and certain gasoline additives^50–52^. But things change. New research casts a valuable light on 2-HIBA, suggesting that it appears to be an important metabolite with potential value for health and disease — 2-HIBA is reported to be up-regulated in known human disorders such as chronic kidney disease^53^, diabetes mellitus^54,55^, human gastric cancer^56^, fibromyalgia^57^, myocardial infarction^58–60^, and a number of inborn errors of metabolism^61,62^. In the present study, alcohol induced significant up-regulation of 2-HIBA in the first two hours after consumption (Table 1: VIP = 1.39; FC = +1.8; *p* = 0.034). The very high correlation of 2-HIBA with lactic acid (r = 0.8), and high correlation with 2-hydroxybutyric and fumaric acids (r = 0.6), strongly supports the view that 2-HIBA is an endogenous metabolite. However, considering the diversity of the conditions associated with the up-regulation of 2-HIBA, a common metabolic origin remains elusive. Most notably, MS analyses of peptide fragments from human histones recently identified a new type of histone mark, namely lysine 2-hydroxyisobutyrylation, which is conserved, widely distributed, has a high stoichiometry, and induces large structural change in histones^63^. Histone acetylation was first identified more than 50 years ago^64^, and has since become known as wide-ranging posttranslational modifications, linked to a variety of processes, including transcription, DNA replication, and DNA damage. A comprehensive catalogue of histone modifications and their proposed functional consequences^65^ includes 2-hydroxyisobutyrylation of lysine side chains, amongst others, for which a rather limited function has been determined. Histone modifications are proposed to present a large opportunity in the years to come to gain insights into chromatin biology, epigenetic events and their biological consequences, which should include the proposed alcoholism-related involvement of 2-HIBA^66^ in the modification of histones^67,68^.

Metabolomics studies, however, have limitations: (1) GC–MS analysis, our method of choice, produces data that are noisier than other metabolomics data types, such as NMR or triple quadrupole liquid chromatography–mass spectrometry (LC–QQQ–MS). This may be overcome by using more experimental subjects (but their number was limited in this study due to ethical considerations), or by using replicates of all samples (rather than only random repeat samples as used in our measurement design). (2) Although it would be useful, quantifying metabolomics data absolutely is often difficult to execute accurately, is relatively expensive, and is rarely done in diagnostic or intervention study designs as presented here. As an acceptable alternative, therefore, we expressed our metabolite concentrations as values relative to an internal standard. (3) Concomitant plasma or serum analyses did not form part of this experimental design. Therefore, the metabolomic variables considered in this investigation were generated from only organic acid analyses of urine samples, and were modelled on theoretical grounds on metabolic pathways of the gut, muscle and liver (Fig. 5). Given the intoxicating influence of alcohol, metabolite levels in the brain are also important, but could also not be considered here due to the biofluid used. However, as baseline, the perturbations of urinary organic acid profiles provide a prime and feasible way for clinical tests to screen for individual response types, as well as for other relevant aspects of acute alcohol exposure.

## Conclusions

Taken together, our findings indicate that metabolomics provided a systematic and standardized method for detecting a range of metabolic responses over time, not previously described comprehensively for acute alcohol consumption. These findings open avenues for potentially important future investigations in alcohol research: (1) genotype-based selection of individuals in follow-up alcohol intervention studies is advised, given the clear inter-individual responses to alcohol consumption; (2) low values of urinary vanillylmandelic acid may be an indicator of a binge drinking or acute alcohol consumption episode in seemingly non-intoxicated individuals; and (3) the striking presence of 2-HIBA supports the emerging new paradigm of 2-HIBA being an important endogenous metabolite. Moreover, detailed studies on the biological origin of 2-HIBA, as well as on its perceived gene-modification role through lysine 2-hydroxyisobutyrylation of histones may take us one step closer to understanding the personalized responses to acute alcohol consumption and the perceived epigenetic changes that are induced. All in all, we concur that acute alcohol consumption studies broaden insights on significant adverse health effects of alcohol even in healthy individuals^42^. These insights will help researchers to define novel approaches to treat or ameliorate alcohol-induced disability, organ damage and morbidity.

## Materials and Methods

### Intervention study design

The experimental group consisted of 12 clinically selected healthy males (aged 20–24 years), who admitted to consuming alcohol at a moderate, social level (baseline alcohol consumption was defined by the participants’ declared levels of drinking). None of the participants used any medication. They were asked to refrain from vitamins, minerals, and other supplementation, and to follow a similar dietary and lifestyle pattern for the duration of the study. The experimental interventions were performed at the Health Clinic of North-West University under controlled conditions. A medical doctor and nurse were present during the period of intervention, and all participants could leave the premises only after approval by the medical doctor. The protocol was approved by the Health Sciences Ethical Committee of North-West University (ethical approval number: NWU-00045-12-S1), conducted in accordance with guidelines for good clinical practice, and all participants provided informed written consent to the research protocol (an example of the informed consent form is included in section 5 of the SI). The protocol was registered as a clinical trial on 3 November 2017 under the Pan African Clinical Trial Registry (registration number: PACTR201711002748255), under the title: *A metabolomics investigation on experimental interventions of acute alcohol consumption*.

The experiments were conducted on two Saturday mornings between 08:00 and 12:00. All participants were required to abstain from alcohol consumption for at least 48 hours preceding the experiment, and to abstain from breakfast on the days of the experiments (that is, to remain in an overnight fasted state). On the first Saturday, half of the participants (randomly selected) were given 500 mL lemon-flavoured sparkling water as vehicle only (contents: fructose and citric acid flavouring; sodium benzoate preservative; sodium cyclamate, aspartame, acesulfame K sweeteners; vitamin C). The other half of the participants received the same quantity of the vehicle, as well as a predefined quantity of alcohol — 1.5 mL alcohol per kilogram body mass^69^. The alcohol used was triple-distilled vodka: 43% alcohol. On the second Saturday, the participants received the alternate intervention to the one received on the first Saturday. On both days the participants were also provided with 1.5 L bottled water, which was the only substance that could be consumed over the 4-hour period of the experiment. Differences in the concentrations of urinary metabolites due to variation in water consumption between the participants were accounted for by determining the creatinine concentration of each sample, and expressing the concentrations of all the quantified metabolites as μmol metabolite/mmol creatinine. Initial (time 0) urine samples were collected just prior to the intervention. Subsequent urine samples were collected at 1, 2, 3 and 4 hours after the start of the experiment. This gave a total of five urine samples from each participant for each intervention. Time 0 urine samples served as controls for each participant, with longitudinal data being compared accordingly.

### Sample handling

After collection, each urine sample was divided into aliquots and stored at −80 °C. Once all the urine samples had been collected, a 1 mL aliquot of each sample was thawed and combined to prepare a pooled quality control (QC) sample. This QC sample was divided into aliquots and once again stored at –80 °C. Another 1 mL aliquot of each urine sample was used for creatinine determination, performed by an external pathology service.

### Metabolomics workflow

**Figure 6:**
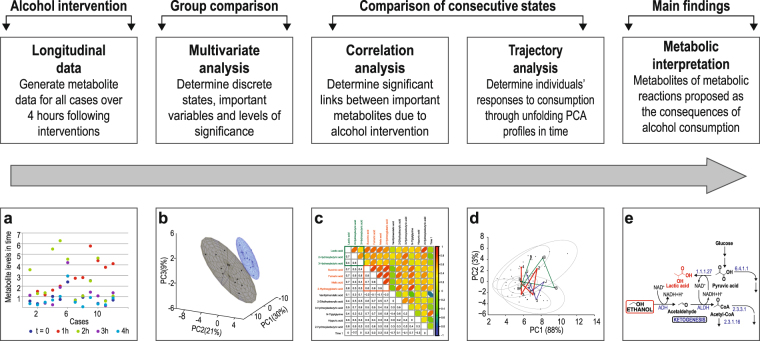
Representation of the metabolomics workflow to investigate the effect of acute alcohol consumption. (**a**) Longitudinal data were generated through the GC–MS analyses for 120 metabolites across five hourly intervals. (**b**) Two metabolic states — before alcohol consumption (time 0) and after alcohol consumption (times 1 to 4 hours) — were compared through multivariate, as well as univariate, analyses to establish the levels of significance of the observed differences. (**c**) Correlation analysis was used to indicate the relationship between important metabolites. (**d**) Unfolded PCA was applied to the data that emerged in time to summarize the subsequent variability, and, more importantly, between individuals, due to the alcohol effect, as well as for the identification of the metabolites responsible for this variability. (**e**) Construction of a global metabolite profile from the combined results provided the framework for the discussion on the effects of acute alcohol consumption on the subjects’ metabolism.

The workflow of the intervention study (Fig. 6) started with the generation of time-dependent quantitative metabolomics data, progressed to the application of various models of statistical analysis, which eventually led to the biological interpretation of the effect of the interventions on the group as well as on individual cases. The samples collected prior to alcohol (or vehicle) consumption were used as controls for the subsequent hourly samples collected after the interventions. The effect of the vehicle-only intervention was previously described^35^, and will not be discussed here.

### Measurement design

For the generation of data through GC–MS analysis, each participant’s samples were analysed in a separate batch. Each batch included the participant’s 10 urine samples (S), repeat samples (R) and quality control samples (Q), and each batch was constructed and analysed as follows:

QQQQQQS_V_S_V_S_V_R_A_QS_V_S_V_R_A_QS_A_S_A_S_A_R_V_QS_A_S_A_R_V_QQ

where:

Q = Quality control sample [Total = 11]

S_V_ = Randomly selected vehicle sample [Total = 5]

S_A_ = Randomly selected alcohol sample [Total = 5]

R_A_ = Randomly selected alcohol repeat sample [Total = 2]

R_V_ = Randomly selected vehicle repeat sample [Total = 2].

QC samples were included to estimate any batch effect or other interfering analytical aspects. The data from the five QC samples at the start of each batch were used to condition the column and were excluded from further analysis. Repeat samples were included to determine their repeatability across the batch — the first two repeat samples in each batch were repeats of experimental samples from later in the batch, and the last two repeat samples were repeats of experimental samples from earlier in the batch. The total GC–MS running time for each batch was approximately 21 hours.

### Organic acid extraction and GC–MS analysis

A 5 mL aliquot of each urine sample was used for organic acid extraction before GC–MS analysis, as described previously^70^. All experimental, QC and repeat samples were prepared and derivatized individually and in the same way. Full details of the method are presented in section 1 of the SI. An excerpt of the data used for the alcohol intervention is shown in Supplementary Table S1 (section 2 of the SI). Metabolite concentrations are expressed as µmol/mmol creatinine relative to an internal standard (4-phenylbutyric acid).

### Quantitative urinary alcohol excretion

Urinary ethanol is not detectable by the GC–MS method used here due to its high volatility, but was quantified in all samples obtained from the vehicle and alcohol interventions by means of NMR spectroscopy. The data indicated that ethanol excretion peaked at 2 hours following alcohol consumption (data not shown).

### Variable identification, classification and reduction

The QC samples were used to identify and classify a list of representative variables in all the urine samples. Following untargeted GC–MS data generation, a total of 172 variables were detected through AMDIS, excluding the 2 internal standards used. Several of the variables were present in concentrations just above the detection limit, and were not observed in all QC samples. The Human Metabolome Database (www.hmdb.ca) was used as the reference for the biological description of each feature, and as the basis for classifying the variables. Information on variables not included in the HMDB (e.g. exogenous substances or artifacts formed during the derivatization reactions) was obtained from other established chemical databases or from the literature; failure with this resulted in the classification of 7 variables as “no annotation”. Details of the 172 variables are summarized in Supplementary Table S2, and exclusion criteria for the determination of metabolite relevance are described in section 3 of the SI. From this protocol, 120 metabolites were identified and used for the statistical evaluation of the effect of alcohol consumption.

### Statistical analysis

#### Identification of important organic acid metabolites

Metabolites causing the separations were regarded as important if they varied substantially between the samples collected prior to alcohol consumption and those collected one hour after alcohol consumption. Changes in metabolite levels were ranked based on their multivariate VIP values (Variable Importance in Projection), fold change (FC) values and non-parametric Wilcoxon signed-rank test (WRT) *p*-values. The selection criteria were: VIP ≥ 1.0, WRT *p* ≤ 0.05 and |FC| ≥ 1.5. The aim with the selection was for a deeper understanding of the dominant biological changes rather than to model the observed data.

### Multivariate statistical analysis

Two modes of multivariate statistical analyses were applied to the metabolomics data generated during this intervention experimental design: (1) cross-sectional analysis of time points using traditional multivariate methods to compare two groups (PCA and PLS-DA); and (2) longitudinal analysis performed across all times (ASCA^34,71^ and unfolded PCA^72^). Details of these methods were previously described^35^.

The statistical analyses indicated that we should achieve acceptable power (0.8) for a large effect size (0.9) given 12 paired observations and a 5% significance level. We should therefore be able to identify large differences between two factor levels (that is, between two points in time or two interventions) for a single variable (see SI section 4.1 for details). Prior to statistical analysis, the data were pre-processed by: (i) treatment of zero-valued observation; and (ii) transformation and scaling. This is explained in greater detail in section 4.2 of the SI. PCA was used to project the observed data to new spaces that maximizes the variation along fewer hyperplanes while not taking the group membership into consideration. PLS–DA was applied to build models to predict group membership, by projecting the variance in the observed data measured and the membership to new spaces. PLS–DA was used as a supervised method to rank and select the metabolites most changed by the intervention. The significance of these changes was established through univariate analysis using the Wilcoxon signed-rank test and fold change ratios. The figures shown in Fig. 1 do not take into account the paired nature of the data. However, multi-level PCA and PLS–DA were also performed, which cater specifically for repeated measures. The multi-level results are closely related to those reported here, and are included in sections 4.3 and 4.5 of the SI.

### Data availability

The full data set is given in Excel format as part of the SI (available online).

## Electronic supplementary material

Supplementary Information

Dataset 1
