# Supplementary material for: GC–MS-based urinary organic acid profiling reveals multiple dysregulated metabolic pathways following experimental acute alcohol consumption
Source: Sci Rep. 2018 Apr 10;8:5775. doi: 10.1038/s41598-018-24128-1 (PMC5893584; doi:10.1038/s41598-018-24128-1)
Supplement: Supplementary file 1 — Supplementary Information [file 41598_2018_24128_MOESM1_ESM.pdf]

GC–MS-based urinary organic acid profiling reveals multiple dysregulated metabolic pathways following experimental acute alcohol consumption — Supplementary Information

Cindy Irwin, Lodewyk J. Mienie, Ron A. Wevers, Shayne Mason, Johan A. Westerhuis, Mari van Reenen, Carolus J. Reinecke

CONTENTS:

1. Sample preparation, organic acid extraction and GC–MS analysis
2. Data used for the alcohol intervention study: Supplementary Table S1 — An excerpt of the data used for the alcohol intervention. The full data set is given as a separate file in Excel format.
3. Details of the 172 identified features: Supplementary Table S2 — Separation and classification details of the 172 identified features
4. Statistical Methods & Additional Results
    - 4.1 Power
    - 4.2 Data pre-processing
    - 4.3 Statistical methods for variable selection
    - 4.4 Correlation analysis
    - 4.5 Statistical methods for repeated measures data
    - 4.6 The vehicle/hippuric acid effect
    - 4.7 Statistical software
5. Example of informed consent form
6. References

1. Sample preparation, organic acid extraction and GC–MS analysis

1.1 Extraction and derivatization of organic acids

The 5 mL aliquots used for the analyses were thawed at room temperature and vortexed before use.

The volume of urine used for the organic acid analysis was based on the creatinine value of each of the samples:

- For creatinine values higher than 100 mg% use 0.5 ml urine
- For creatinine values less than 100 mg% but higher than 5 mg% use 0.5 ml urine
- For creatinine values less than 5 mg% but higher than 2 mg% use 2 ml urine
- For creatinine values less than 2 mg% use 3 ml urine.

The correct volume of urine was transferred to a 6 ml Kimax tube and 6 drops of 5M HCl was added to adjust the pH of the urine to below 2.

The volume of internal standard (IS) added was determined by multiplying the creatinine value (in mg%) with five times the volume of urine used. A mix of malonic acid (RT ~14 minutes) and 4-phenylbutyric acid (RT ~20 minutes) was used as internal standard. The IS was prepared by dissolving 52.5 mg of each in 50 ml milli-Q water. The solution was sonicated for 30 minutes to ensure that all compounds were properly dissolved. 4-Phenylbutyric acid was used owing to its absence in normal urine and in known pathological conditions. In addition, it elutes almost in the middle of the organic acid profile and theoretically co-elutes with very few, if any, other organic acids. Malonic acid was added as a second IS reference peak in the GC chromatogram.

The first extraction was done by adding 6 ml of ethylacetate to each of the samples. The tubes were capped and checked for leakage by inverting the tubes before being mixed on a rotary wheel for 30 minutes. The samples were then centrifuged at 3000 rpm for 3 minutes and the organic phase (top phase) was aspirated into a clean 6 ml Kimax tube.

The second extraction was done by adding 3 ml of diethylether to the aqueous phase (lower phase) and the tubes were once again capped and checked for leakage. The samples were then mixed on the rotary wheel for 10 minutes and centrifuged at 3000 rpm for 3 minutes. The organic phase was aspirated and added to the ethylacetate phase and the aqueous phase was discarded.

Approximately 2 ml of anhydrous sodium sulphate (Na_2SO_4) was added to each sample and the tubes were capped and vortexed to mix. Proper dispersion of the Na_2SO_4 ensures that all the water is removed from the organic phase. The samples were then centrifuged at 3000 rpm for 1 minute and the organic phase was decanted into a clean 3 ml Kimax tube. The samples were finally evaporated

to dryness in a heating block at 37ºC under nitrogen gas for approximately 45 minutes.

Using a Hamilton syringe, the derivatization reagents O-bis(trimethylsilyl)trifluoroacetamide (BSTFA), trimethylchlorosilane (TMCS) and pyridine were added to the dried samples. The volume of BSTFA added was determined by multiplying the creatinine value (in mg%) with three times the volume of urine used. The volume of TMCS and pyridine added was determined by multiplying the creatinine value (in mg%) with 0.6 times the volume of urine used. The tubes were capped and incubated at 70ºC for 45 minutes.

The derivatized samples were then transferred to 1.5 ml GC–MS vials with inserts, capped, and placed in the GC–MS autosampler for GC–MS analysis. Blank (hexane), QC and repeat samples were included in the injection sequence, as described in the main text.

The internal standards 4-phenylbutyric acid and malonic acid as well as the anhydrous Na_2SO_4 were purchased from BDH. Ethylacetate and dietylether were obtained from Merck Chemicals. The derivatization reagents O-bis(trimethylsilyl)trifluoroacetamide (BSTFA), trimethylchlorosilane (TMCS) and pyridine were purchased from Sigma Chemical Company.

1.2 GC–MS analysis

An Agilent GC–MS system was used in this study and consisted of a model 7890A gas chromatograph, a model 5975C mass selective detector, a Hewlett Packard 5970C quadrupole mass spectrometer and Agilent Chemstation. GC separation was achieved on a WCOT fused silica capillary column [30 m x 0.32 mm (i.d.)] coated with SE30 CB, film thickness 0.30 µm (Machery Nagel).

Samples were introduced into the column via a splitless injector at 280°C. The initial oven temperature was kept at 60°C for 2 minutes and then programmed to rise 6°C/min to a final temperature of 280°C. This temperature was maintained for 5 minutes. Helium was used as carrier gas at a pressure of 60 kPa and at a constant flow rate of 1 ml/min. The column was inserted directly into the ion source with an interface temperature of 280°C. The mass spectra of all GC peaks were generated by a mass spectrometer operated in the electron impact (EI) mode, with electron energy of 70 eV. The MS source and quadrupole temperatures were 250°C and 150°C respectively.

1.3 Qualitative analysis of metabolites in QC samples (metabolite identification)

After GC separation, each peak at an indicated RT was analysed in EI mode in order to investigate the fragmentation pattern of each compound. The m/z values selected for feature identification were either the base peak ion or one of the more abundant characteristic fragment ions.

Deconvolution and data analyses were conducted using AMDIS software (Version 2.71) linked to NIST Mass Spectral Search Program for the NIST/EPA/NIH Mass Spectral Library (Version 2.0F, built Oct. 8, 2008). Where authentic standards were available, their respective response factors were used, and for those compounds where no authentic standards were available, a response factor of 1 was assumed. The analytical setting of the AMDIS software was as follows: minimum factor – 60%, threshold – “off”, Scan Direction – “high to low”, and type of analysis – ‘‘Use of an internal standard for RI’’. The deconvolution settings were: component width – 12, adjacent peak subtraction – 1, resolution – medium, sensitivity – low, and shape requirements – low. The first hit of identified compounds and integrated area of the peaks were exported to Microsoft Excel.

2. Data used for the alcohol intervention study

Supplementary Table S1 shows an excerpt of the data used for the alcohol intervention study (the full data set is given as a separate file in Excel format).

Supplementary Table S1 An excerpt of the data used for the alcohol intervention study

		1	2	3	4	5	6	7	8	9	…	119	120
**Batch**	**Time**	**Glyoxylic-**	**1,2-Dihyd**	**1,2-Dihyd**	**Lactic-aci**	**Hexanoic-**	**2-Hydroxy**	**Glycolic-a**	**Oxalic-aci**	**2-Hydroxy**		**Octadecar**	**Eicosanoi**
1	0	0.181	0.825	6.424	8.188	0.198	8.959	7.566	2.127	3.362		1.918	0.030
1	1	0.004	0.863	0.586	6.011	0.194	7.354	3.867	1.043	1.983		1.086	0.035
1	2	0.000	3.348	23.576	73.513	1.017	37.804	11.328	1.472	5.640		0.125	0.014
1	3	0.004	1.715	2.798	14.757	0.219	11.194	1.895	1.719	2.231		5.534	0.057
1	4	0.004	1.544	3.411	16.042	0.525	12.337	3.209	0.507	1.175		3.472	0.012
2	0	0.380	6.090	3.299	10.635	0.213	13.432	5.542	1.656	2.546		1.380	0.006
2	1	0.577	6.087	5.495	31.238	0.207	51.693	12.475	3.070	8.968		7.982	0.015
2	2	2.431	10.583	6.763	19.799	0.040	24.936	3.507	4.564	4.293		16.834	0.064
2	3	0.165	6.804	2.695	8.701	0.031	13.519	1.426	2.046	2.385		3.687	0.016
2	4	0.392	5.628	0.717	11.225	0.016	22.899	4.268	1.535	3.195		2.861	0.018
3	0	0.057	6.082	1.117	5.128	0.017	7.560	2.057	0.249	1.832		0.587	0.002
3	1	0.263	6.834	1.312	6.123	0.005	11.984	2.682	0.708	2.046		0.579	0.008
3	2	0.074	7.555	3.482	11.516	0.041	10.888	1.091	0.437	0.051		4.187	0.009
3	3	0.009	7.164	0.762	2.578	0.011	4.549	0.295	0.566	0.173		1.701	0.000
3	4	0.142	7.788	2.470	7.058	0.023	10.243	1.352	2.480	0.027		5.931	0.012
4	0	1.260	5.450	14.115	13.301	0.008	19.194	3.939	1.727	3.589		1.668	0.004
4	1	0.786	6.998	4.797	7.539	0.005	11.048	1.319	0.234	0.600		2.648	0.019
4	2	5.384	8.793	7.255	22.786	0.074	34.901	1.477	1.401	2.113		20.830	1.148
4	3	0.452	6.635	2.458	3.065	0.002	6.041	0.249	0.109	0.308		1.754	0.022
4	4	10.047	5.740	14.662	20.996	0.176	32.883	3.912	0.401	2.180		5.673	0.038
5	0	0.190	5.314	1.160	13.823	0.059	18.080	6.760	1.977	3.394		1.605	0.040
5	1	0.348	5.662	2.762	16.770	0.036	20.638	8.213	2.311	4.511		3.696	0.830
5	2	0.004	13.903	16.727	58.947	0.143	44.790	9.347	11.309	9.393		20.223	0.187
5	3	0.041	9.541	9.511	52.548	0.150	29.792	5.183	1.630	3.260		21.992	0.056
5	4	3.452	6.023	1.227	12.596	0.041	19.668	4.064	1.310	1.860		5.391	0.045

3. Details of the 172 identified features

Supplementary Table S2 gives the details of each of the 172 identified and classified variables. A reference value for the normal concentration range in urine for adults of each identified variable is also given.

Supplementary Table S2 Identification, classification and reference ranges of 172 identified variables

	Tentative variable name based on AMDIS and the library used	RT (min)	Variable no.	Index reference	Reference range (μmol/mmol Cr)	Excluded variable and criteria
1	Glyoxylic acid	7.769	1	HMDB00119	1.27 (0.46–3.26)	
2	2-Undecenoic acid	8.029		CS 21171839	n/r	Not a recognized biological substance
3	1,2-Dihydroxyethane	8.693	2	KEGG C01380	n/r	
4	1,2-Dihydroxypropane	9.211	3	ChEBI 16997	n/r	
5	Boric acid	9.24		HMDB35731	n/r	Not a recognized biological substance
6	Phenol	10.158		HMDB00228	4.8 (0.6–12.8)	Not a recognized biological substance
7	O-acetylphenol	10.323		HMDB32568	n/r	Not a recognized biological substance
8	Lactic acid	10.652	4	HMDB00190	3.9–9.8	
9	Hexanoic acid	10.75	5	HMDB00535	n/q	
10	2-Hydroxyisobutyric acid	10.881	6	HMDB00729	4.4–7.6	
11	Glycolic acid	1.952	7	HMDB00115	64.0 (21.0–107.0)	
12	Oxalic acid	12.229	8	HMDB02329	27.00 (0.00–54.00)	
13	2-Hydroxybutyric acid	12.549	9	HMDB00008	2.8 (1.2–6.9)	
14	4-Ketovaleric acid	12.603	10	HMDB00720	1.3 (0.4–2.0)	
15	4-Cresol	12.834		HMDB01858	46.0 (1.2–118.9)	Questionable biological substance/origin
16	3-Hydroxypropionic acid	12.882	11	HMDB00700	8.1 (3.1–11.8)	
17	2-Methyl-2-hydroxybutyric acid	12.921	12	HMDB01987	0.8 (0.4–1.5)	
18	Sulphate	12.954		HMDB01448	2407.89 (2171.05–2697.36)	Not a recognized biological substance
19	2-Hexenoic acid	13.057	13	HMDB10719	n/r	
20	3-Hydroxybutyric acid	13.421	14	HMDB00357	1.4–2.2	
21	3-Hydroxyisobutyric acid	13.442	15	HMDB00023	11 (4.1–19)	
22	2-Hydroxyisovaleric acid	13.665	16	HMDB00407	0.23–0.42	
23	N-Crotonylglycine	13.82	17	CAS 71428-89-2	n/r	Glycine conjugation product of crotonic acid
IS	*Malonic acid*	14.402	IS-2			
24	2-Methyl-3-hydroxybutyric acid	14.518	18	HMDB00354	4.2 (1.6–6.7)	
25	Methylmalonic acid	14.74	19	HMDB00202	1.8 (0.00–3.6)	
26	3-Hydroxyisovaleric acid	14.802	20	HMDB00754	11 (6.9–25)	
27	Benzoic acid	15.208		HMDB01870	4.2 (1.9–6.5)	Preservative of the vehicle
28	2-Ethyl-3-hydroxypropionic acid (2-ethylhydracrylic acid)	15.277	21	HMDB00396	2.1 (1.3–2.9)	
29	3-Hydroxyvaleric acid	15.462	22	HMDB00531	1 (0–2)	
30	Acetoacetic acid	15.532	23	HMDB00060	0.15 (0.01–0.58)	
31	2-Methyl-3-ketobutyric acid	15.601	24	HMDB03771	1.0 (0.0–2.0)	
32	4-Pyridinecarboxylic acid	15.741		HMDB60665	n/r	Derivatization artifact
33	Octanoic acid	15.899	25	HMDB00482	n/q	
34	2,2-Dihydroxyacetic acid	15.929		CS 2005843	n/r	Not a recognized biological substance

	Tentative variable name based on AMDIS and the library used	RT (min)	Variable no.	Index reference	Reference range (μmol/mmol Cr)	Excluded variable and criteria
35	2-Ketoisovaleric acid	15.6	26	HMDB00019	0.13 (0.00–0.54)	
36	N-Acetylglycine	15.66	27	HMDB00532	n/r	
37	Phosphoric acid	16.288	28	HMDB02142	784 (425–1170)	
38	Octanoic acid	15.899	29	HMDB00482	n/q	
39	3-Ketovaleric acid	16.198	30	CS 388751	n/r	Can be of endogenous origin in certain metabolic disorders
40	Phenylacetic acid	16.37	31	HMDB00209	0.3 / 1.9	
41	Ethylmalonic acid	16.379	32	HMDB00622	1.16–2.99	
42	Glycerol	16.687	33	HMDB00131	13 (4–19)	
43	2-Octenoic acid	16.954	34	HMDB00392	n/r	
44	Succinic acid	17.024	35	HMDB00254	9.9 +/- 5.0	
45	2-Hydroxyglutaryllactone	17.141		n/a	n/r	From 2-hydroxyglutaric acid (see no. 104)
46	3-Hydroxycaproic acid	17.169	36	HMDB02203	n/r	
47	1,2-Dihydroxybenzene	17.263	37	HMDB00957	4.06 +/- 1.80	
48	Methylsuccinic acid	17.382	38	HMDB01844	2.3 (0.8–10.8)	
49	Pyrimidinedione	17.48		n/a	n/a	Derivatization artifact
50	Uracil	17.555	39	HMDB00300	9.5 (2.6–22.8)	
51	5-Hydroxyvaleric acid	17.52	40	HMDB61927	n/r	
52	Citraconic acid	17.81		HMDB00634	1.6 (0.9–2.1)	Not a recognized biological substance
53	Glyceric acid	17.815	41	HMDB00139	1.7 (0.2–6.0)	
54	Glutaconic acid	17.839	42	HMDB00620	3.1 (1.2–3.1)	
55	Fumaric acid	17.889	43	HMDB00134	0.75–1.2	
56	Nonanoic acid	18.115	44	HMDB00847	n/r	
57	5-Hydroxyhexanoic acid	18.224	45	HMDB00525	2.7 (0.8–5.7)	
58	N-Isobutyrylglycine	18.297	46	HMDB00730	n/q	
59	2,3-Dihydroxybutanoic acid	18.391	47	HMDB00498	5.0 +/- 3.0	
60	Lactyllactate	18.762		CS 91775	n/r	From lactic acid
61	1,3-Dihydroxybenzene	18.62		HMDB32037	n/r	Questionable biological substance
62	Hydroxymalonic acid	18.913		HMDB35227	n/r	Derivative from IS-2
63	Glutaric acid	18.975	48	HMDB00661	1.3 (0.6–2.6)	
64	Phenoxyacetic acid	19.083		HMDB31609	n/r	Questionable biological substance
65	Benzamide	19.153		HMDB04461	n/r	Not a recognized biological substance
66	2,3,4-Trihydroxybutyric acid-lactone	19.32		n/a	n/r	From 2,3,4-trihydroxybutyric acid
67	N-Butyrylglycine	19.46	49	HMDB00808	0.24 +/- 0.36 [infant]	
68	3-Methylglutaric acid	19.442	50	HMDB00752	1.0–6.5	
69	N-Isobutyrylglycine(2)	19.46		HMDB00730	n/q	Second peak (see no. 58)
70	Malonic acid-triTMS	19.602		Agilent G1676AA	n/r	Derivative of IS-2
IS	*4-phenylbutyric acid*	19.636	IS-1			
71	3-Methylglutaconic acid	19.698	51	HMDB00522	6.2 (2.8–8.3)	
72	3-Hydroxyadipyllactone	19.748	52	n/a	n/r	From adipic acid (see no. 86)
73	N-Acetylleucine	19.768	53	HMDB11756	n/r	
74	3,4-Dihydroxybutyric acid	19.952		HMDB06118	34.9 (12.5–57.2)	Questionable biological substance
75	Decanoic acid	20.1	54	HMDB00511	n/q	
76	N-Isovalerylglycine	20.22	55	HMDB00678	2.0 (0.4–4.0)	
77	Parabanic acid	20.308		CID 67126	n/r	Medication/Artifact

	Tentative variable name based on AMDIS and the library used	RT (min)	Variable no.	Index reference	Reference range (μmol/mmol Cr)	Excluded variable and criteria
78	2,2-Dihydroxymalonic acid	20.473		CS 61695	n/r	Derivative from IS-2
79	3-Ethylglutaric acid	20.54	56	n/a	n/a	Potential ethanol derivative
80	5-Methyldihydropyrimidine-2,4(1H,3H)-dione	20.572		CS 84456	n/r	Not a recognized biological substance
81	3,5-Dihydroxy-3-methylvaleric acid	20.697	57	HMDB00227	0.14 (0.06–0.22)	
82	N-Isovalerylglycine(2)	20.823		HMDB00678	2.0 (0.4–4.0)	Second peak (see no. 76)
83	Pyroglutamic acid	21.128	58	HMDB00267	14.0 +/- 7.2	
84	3-Methylglutaconic acid	20.626	59	HMDB00522	6.2 (2.8–8.3)	Second peak (see no. 71)
85	Citramalic acid	20.717		HMDB00426	2.4 (1.0–4.8) [NMR analysis]	Questionable biological substance
86	Adipic acid	20.94	60	HMDB00448	5.1 (0.8–35)	Can be of endogenous origin in certain metabolic disorders
87	Malic acid	20.942	61	HMDB00156	2.0 (0.7–5.3)	
88	2-Deoxy-3,5-dihydroxypentonic acid-g-lactone	20.971	62	n/a	n/a	
89	2-Piperidinecarboxylic acid	21.14		HMDB00070	0.28 (0.0–0.56)	Questionable biological origin
90	4-Hydroxycyclohexylcarboxylic acid	21.243		HMDB01988	6.1 (1.1–28.1) [Adolescent]	Questionable biological substance
91	5-Hydroxyhydantoin	21.323		CID 4157426	n/r	Derivatization artifact
92	N-Acetylthreonine	21.356	63	CS 3841173	n/r	
93	3-Methyladipic acid	21.518	64	HMDB00555	2.9 (0.7–10.5)	
94	5-(Hydroxymethyl)furan-2-carboxylic acid	21.651		HMDB02432	1.7	Questionable biological substance
95	2,4-Hexadienedioic acid	21.65		HMDB29581	n/r	Questionable biological substance
96	2-Hydroxyphenylacetic acid	22.081	65	HMDB00669	2.0 (0.9–4.5)	
97	N-Tiglylglycine	21.736	66	HMDB00959	0.78–1.2	
98	Threitol	21.744	67	HMDB04136	19.3 (5.3–32.7)	
99	2-Hydroxy-2-methylglutaric acid	21.901	68	Yancey *et al.*, 1986	n/r	
100	2-Hydroxyphenylacetic acid	22.081	69	HMDB00669	2.0 (0.9–4.5)	
101	N-3-Methylcrotonylglycine	22.128	70	HMDB00459	1.0 (0.0–2.0)	
102	2,3,4-Trihydroxybutyric acid	22.275		HMDB00613	20.0 +/- 8.0	Questionable biological substance
103	N-2-Pentenoylglycine	22.358	71	n/a	n/a	
104	2-Hydroxyglutaric acid	22.453	72	HMDB00694	0.8–52	
105	3-Hydroxyglutaric acid	22.49	73	HMDB00428	<11.51 [children]	
106	2,3,4-Trihydroxybutyric acid(2)	22.52	74	HMDB00613	20.0 +/- 8.0	Second peak (see no. 102)
107	2,4,6-Trihydroxypyrimidine	22.665		HMDB41833	n/r	Derivatization artifact
108	Pimelic acid	22.678	75	HMDB00857	2.2 (0.7–4.0)	
109	Hexahydropyrimidine-2,4,5-trione	22.696		n/a	n/a	Not a recognized biological substance
110	3-Ketoglutaric acid	22.841	76	HMDB13701	0–0.11	
111	N-Hexanoylglycine	22.869	77	HMDB00701	1.00 (0.0–2.0)	
112	2-Ketoglutaric acid	22.949	78	HMDB00208	2.87 (0.18–14.3)	
113	3-Hydroxyphenylacetic acid	22.753	79	HMDB00440	0.6 (0.4–0.9)	
114	3-Hydroxy-3-methylglutaric acid	22.992	80	HMDB00355	3.2 (1.1–5.2)	
115	4-Hydroxycyclohexylacetic acid	23.047		HMDB00909 (trans)	n/r	Not a recognized biological substance. Alternative reference: CS 13628091 (cis)

	Tentative variable name based on AMDIS and the library used	RT (min)	Variable no.	Index reference	Reference range (μmol/mmol Cr)	Excluded variable and criteria
116	4-Hydroxybenzoic acid	23.143	81	HMDB00500	1.8 (0.7–29)	
117	4-Hydroxyphenylacetic acid	23.249	82	HMDB00020	6.0 (2.4–9.7)	
118	2,5-Furandicarboxylic acid	23.255		HMDB04812	1.9 (0.1–5.4) [Adolescent]	Derivatives found in food prepared by strong heating. Not a recognized biological substance
119	N-Acetylaspartic acid	22.18	83	HMDB00812	4.66 +/- 1.14	
120	2,3,4,5-Tetrahydroxypentanoic acid-1,4-lactone	23.496	84	CAS 179091-67-9	n/r	From ascorbic acid (see no. 151)
121	Dodecanoic acid	23.807	85	HMDB00638	0.03 +/- 0.02	
122	2-Furanylcarbonylglycine	23.639		HMDB00439	9.95 (2.00–18.66)	Not a recognized biological substance (see no. 118)
123	N-Cyclohexylsulfamic acid	23.849		HMDB31340	n/r	Not a recognized biological substance
124	N-Acetylanthranilic acid	23.908	86	CID 6971	n/r	
125	Octenedioic acid	23.897	87	HMDB00341	n/r	
126	Isocitric acid-lactone	24.048		CID 98259	n/r	From isocitric acid (see no. 145)
127	Suberic acid	24.255	88	HMDB00837	0.5 (0.0–2.9)	
128	Erythro-pentonic acid	24.561	89	n/a	n/r	Possible CAS 74742-30-6
129	Acetyl-4-phenol	24.595		CS 7189	n/r	Not a recognized biological substance
130	Tricarballylic acid	25.195		HMDB31193	n/r	Derivatization artifact
131	Citric acid-diethylester	25.195	90	CAS 19958-02-2	n/r	
132	Aconitic acid	25.195	91	HMDB00461	13 (2.7–44)	
133	Vanillic acid	25.283	92	HMDB00484	1.0 (0.0–2.5)	
134	Homovanillic acid	25.347	93	HMDB00118	5.6 (2.1–47.3)	
135	Pyrrole-2-carboxylic acid	25.551	94	HMDB03094	0.03 +/- 0.02	
136	2,5-Dihydroxybenzoic acid	25.686	95	HMDB00152	1.35 +/- 0.79	
137	4-Hydroxymandelic acid	25.737	96	HMDB00822	0.98–1.5	
138	Azelaic acid	25.819		HMDB00784	4.8 (1.3–15)	Questionable biological substance
139	Hippuric acid-diTMS	26.055		CS 454967	n/r	Derivative of hippuric acid
140	Citric acid-monoethylester	25.923	97	CID 57345948	n/r	
141	Hippuric acid-TMS	26.058	98	HMDB00714	27.92–932.66	
142	3,5-Dihydroxybenzoic acid	26.281	99	HMDB13677	0.218 +/- 0.124	
143	3,4-Dihydroxybenzoic acid	26.34		HMDB01856	0.048 +/- 0.008	Questionable biological substance
144	3,4-Dihydroxyphenylacetic acid	26.454	100	HMDB01336	0.9 (0.6–1.3)	
145	Isocitric acid	26.552	101	HMDB00193	56.8 (19.4–119.1)	
146	Citric acid	26.584	102	HMDB00094	172.8 +/- 87.4	
147	Tetradecanoic acid	26.65	103	HMDB00806	0.06 +/- 0.03	
148	3-Hydroxyphenylhydracrylic acid	26.844		HMDB02643	5.9 (1.4–22.1)	Questionable biological substance
149	Methylcitric acid	27.008	104	HMDB03610	0.59 (0.05–1.15)	
150	Vanillylmandelic acid	27.168	105	HMDB00291	1.1–1.7	
151	Ascorbic acid	27.303		HMDB00044	32.5 (4.6–78)	Vehicle additive
152	Hydantoinpropionic acid	27.255		HMDB01212	n/r	Questionable biological substance
153	4-Hydroxyphenyllactic acid	27.571	106	HMDB00755	1.1 (0.2–2.6)	
154	1H-Indole-3-acetic acid	28.028	107	HMDB00197	1.5–2.6	
155	3,4-Dihydroxyphenylpropionic acid	28.131		HMDB00423	0.27 +/- 0.079	Questionable biological substance

	Tentative variable name based on AMDIS and the library used	RT (min)	Variable no.	Index reference	Reference range (μmol/mmol Cr)	Excluded variable and criteria
156	3-(4-Hydroxy-2,5-dioxoimidazolidin-4-yl) propanoic acid	28.155		n/a	n/a	Not a recognized biological substance
157	3-Methoxy-4-hydroxyphenylhydracrylic acid	28.761		Smith, 1969	n/r	Not a recognized biological substance
158	2,4,6-Trihydroxybenzoic acid	28.771		CID 66520	n/r	Not a recognized biological substance
159	Palmitic acid	29.416	108	HMDB00220	11 (6.0–23)	
160	2-Hydroxyhippuric acid	29.501	109	HMDB00840	0.5	
161	Glucuronic acid	29.734	110	HMDB00127	9.7 (3.7–20.6)	
162	4-Methoxy-3-hydroxycinnamic acid (Isoferulic acid)	29.886	111	HMDB00955	0.394 +/- 0.075	
163	N-Acetyltyrosine	29.886	112	HMDB00866	<10 [children]	
164	4-Hydroxyphenylpyruvic acid	29.738	113	HMDB00707	1.65 (0.15–8.74)	
165	Heptadecanoic acid	30.693	114	HMDB02259	n/r	
166	N-Cinnamoylglycine	30.723	115	HMDB11621	n/r	
167	4-Hydroxyhippuric acid	31.15	116	HMDB13678	0–14	
168	Phenylacetylglutamine	30.71	117	HMDB06344	47.03 (3.84–85.51)	
169	4-Hydroxyhippuric acid(2)	31.423		HMDB13678	0–14	Second peak (see no. 167)
170	Oleic acid	31.583	118	HMDB00207	5.2 (0.3–13)	
171	Octadecanoic acid	31.904	119	HMDB00827	2.9 (1.6–6.6)	
172	Eicosanoic Acid	33.85	120	HMDB02212	n/r	

The original list of 172 variables contained substances that were excluded from the metabolomics analysis to assess the influence of acute alcohol consumption. Only the 120 included substances are numbered in column 4 of the table. The reasons for the exclusion of the other 52 substances are given in the final column of the table.

Categories for exclusion of variables were:

1. Exogenous contaminants that are not recognized biological substances.
2. Substances of a questionable biological function or origin, based on literature assessments.
3. Artifacts formed from chemicals (e.g. urea) present in the urine.
4. Artifacts due to the reaction conditions (e.g. formation of lactones, used for correction of the parent substance).
5. Artifacts due to formation of an additional TMS derivative (used for correction of the parent substance).
6. Substances showing multiple peaks in the GC-profile (used for correction to only one substance).

4. Statistical Methods & Additional Results

4.1 Power

Only a limited number of subjects could be included in an intervention study of this nature, i.e. exposure to a substance (alcohol) with known health risks. It was therefore necessary to ensure that the small sample size would not prevent the quantification of the effect of the intervention. A power calculation was performed assuming the following:

(i) The effect of the intervention would be large, with an expected effect size of at least 0.8 when calculated based on Cohen's d-value[1]. This was considered a valid assumption given the design of the study, i.e. consuming a relatively large volume of alcohol on an empty stomach.

(ii) The dependence between samples from the same individual would be taken into account by using the dependent samples, or paired t-test, to assess the significance of the effect of the intervention. Though this method is a parametric univariate method comparing only two groups, it was deemed appropriate as we could transform the data to achieve normality, we did not know beforehand how many metabolites would be extracted, and the most dramatic effect of the intervention was expected to be between time 0 (before consumption of the alcohol) and 1 hour after consumption based on a pilot study.

(iii) A significance level of 5% for a two-tailed test and a sample size of 12. We did not want to speculate on the metabolites that would be extracted and hence could not speculate on the direction of the expected change. The two-tailed hypothesis was therefore selected.

The power under these conditions is plotted against the effect size in Supplementary Fig. S1, from which it is evident that power values above 0.7 are achieved for effect sizes of 0.8 or higher. This was acceptable in the current context. The power curve given a one-sided hypothesis is also included for reference purposes to show the increased power.

a

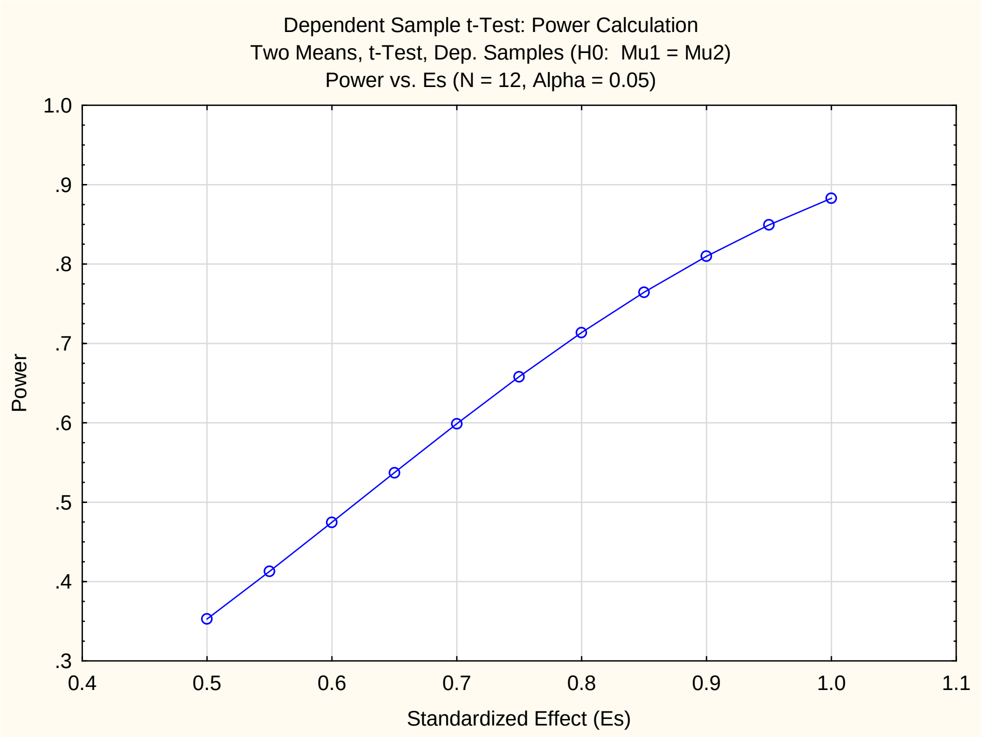

b

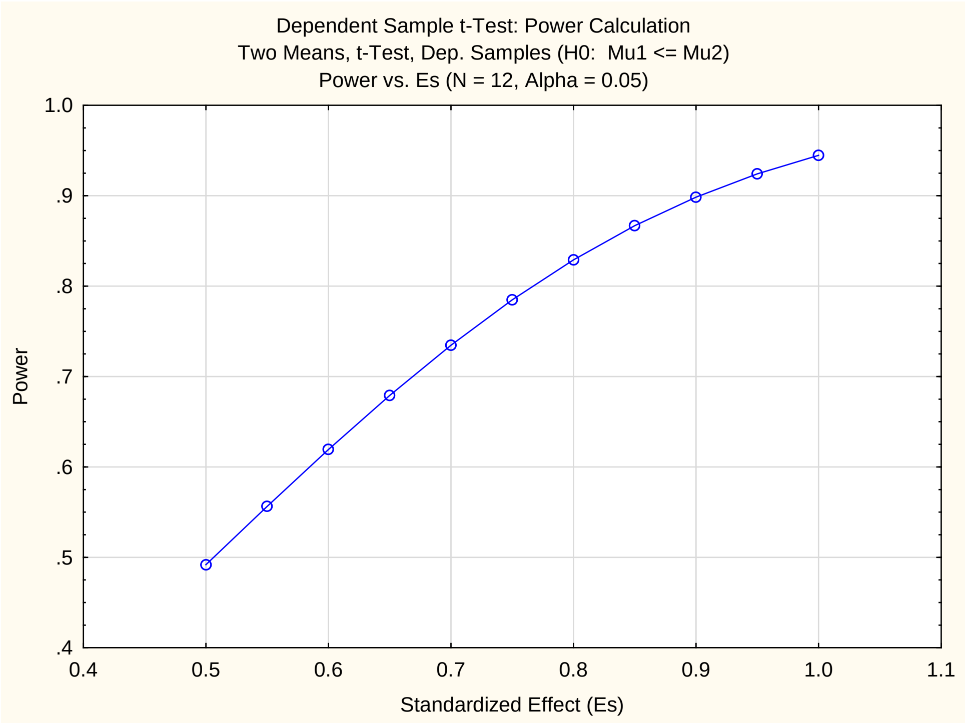

Supplementary Figure S1. Power curves

Power curves for a dependent samples t-test for a sample size of 12 and a 5% significance level, when setting a two-sided (a) and one-sided (b) alternative hypothesis.

4.2 Data pre-processing

The collection and analysis of biological samples culminated in 12 subjects observed across 5 time points and measured over 120 metabolites. A 50% zero filter was applied, taking time into account — that is, variables were excluded if they contained more than 50% zero-values in all 5 times. Two variables were removed based on these criteria. The remaining zero-values were imputed for each variable by randomly generating numbers from the tail of beta-distribution fitted to the non-zero observations. The resulting random numbers were all smaller than half of the minimum observed value. The data were then log transformed and centred prior to further statistical analysis.

4.3 Statistical methods for variable selection

It was decided to deconstruct the three-dimensional data tensor into less complex cross-sections in order to select a subset of statistically important metabolites to aid biological interpretation of the observed perturbations. The assumption was made that the most biologically acute effect of the intervention would peak one hour after alcohol consumption. The cross-section between time 0 (before consumption of the alcohol) and one hour after consumption was therefore analysed using univariate and multivariate statistical methods.

The univariate nonparametric Wilcoxon signed-rank test was used to assign significance levels (p-values) to changes in metabolite levels from time 0 to time 1. The dependent t-test was also applied, after data transformation to ensure normality, but not used as current research revealed that the Wilcoxon signed-rank test to be a suitable alternative with marginally more power for a non-normally distributed data set of smaller sample size[2].

Multivariate partial least squares-discriminant analysis (PLS–DA) is a method that constructs a regression model to predict group membership by projecting variance in the metabolites measured, as well as in the observed group membership, to new spaces. PLS–DA models can easily overfit and produce models with inflated predictive ability unless extensively validated through test data and permutation testing. For this reason, the PLS–DA results are used cautiously as the small sample size did not allow for proper validation. The results used are limited to the VIP values, which are produced by the PLS–DA model for each metabolite as an indication of its predictive strength. Given our concern here, it is again important to emphasize that the aim of this selection was not to model the observed data, but rather to gain a deeper understanding of the predominant biological changes occurring due to the intervention. Ranking and selecting metabolites according to VIP values enabled us to identify metabolite combinations which dominated the observed change in metabolomic state. Ultimately, we revert back to the raw data (means and standard deviations) to interpret the resulting list against established metabolic pathways, and including proposals for extension or modification of them, resulting in a

representation of a global metabolite profile reflecting the metabolic consequences of the alcohol consumption.

PCA is similar to PLS–DA in that observed data are also projected, but to new spaces that maximize variation along fewer hyperplanes, not taking the group membership into consideration.

A subset of metabolites was subsequently identified to gain a deeper understanding of the predominant biological changes occurring due to the intervention. The selection was not made for the purpose of modelling the effect of the intervention in time nor to predict group membership. Changes in metabolites levels were then ranked based on their multivariate VIP values. The significance of changes in high-ranking variables was then established through fold change (FC) values and non-parametric Wilcoxon signed-rank test (WRT) *p*-values. The selection criteria were then as follows: VIP ≥ 1.0, WRT $p \le 0.05$ and $|FC| \ge 1.5$.

4.4 Correlation analysis

Associations between metabolites shortlisted for biological interpretation were assessed using Spearman's rho or rank order correlation coefficient. This method is nonparametric as this takes the ranks of the data as inputs. The correlation coefficients (r-values) produced are seen as biologically relevant if $|r| \ge 0.5$[1].

4.5 Statistical methods for repeated measures data

The data tensor evaluated here has a very specifically designed structure with the same individuals being measured over and over, hence a repeated measures design over 5 points in time. Although the Wilcoxon signed-rank test accounts for groups of data not being independent but paired, standard PCA and PLS–DA applications do not. To understand the importance of this information for these models, a multi-level PCA and PLS–DA on the within-subject variation was also performed.

Supplementary Fig. S2 provides the scores plots corresponding to the cross-sections of the data used in the main text. The PCA scores plots are comparable with those included in the main text, showing some differentiation one hour after the alcohol consumption (Fig. S2A), followed by complete separation after 2 and 3 hours. The separation is, however, retained after 4 hours in the multi-level PC model. That said, a slight yet progressive return after 3 and 4 hours to the profile at time 0 is still evident when considering the decreasing variance explained by the first principal component. The PLS–DA plots again showed a complete separation for all four times following alcohol consumption relative to time 0.

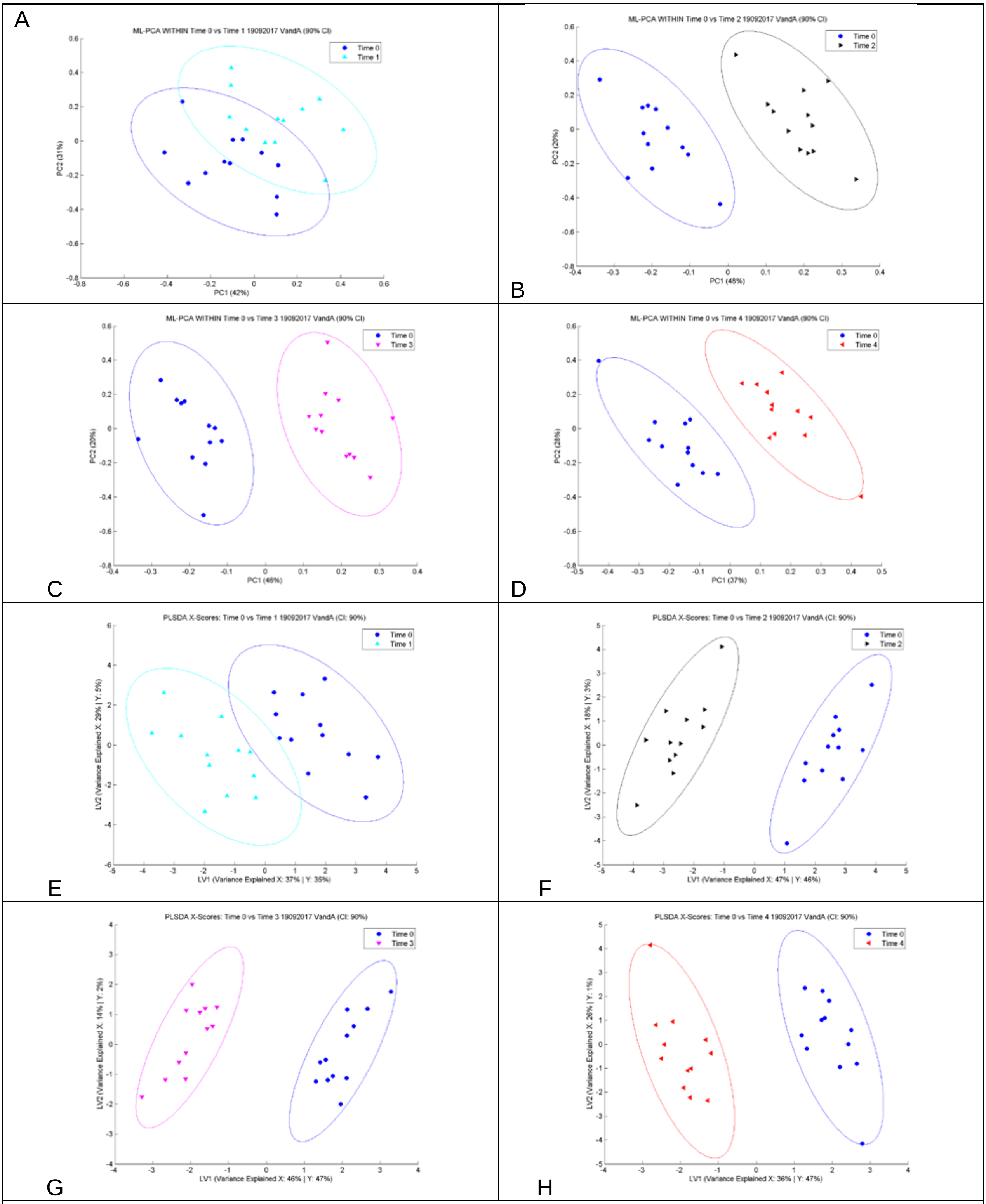

Supplementary Figure S2. PCA and PLS–DA plots following alcohol consumption. Input data were from the 120 quantified metabolites for time 0 vs time 1 (A & E), time 0 vs time 2 (B & F), time 0 vs time 3 (C & G), and time 0 vs time 4 (D & H) following alcohol intake. The samples for time 0 were collected just prior to the interventions and are therefore regarded as the control samples.

Finally, the entire data tensor was modelled using PCA. This was achieved by unfolding the data along the time dimension[3].

This approach allows for the projection of data to fewer dimensions, while retaining the maximum amount of variation and understanding of the changes observed in time. This can then be observed and compared over all individuals, by averaging scores, or for each individual to allow for comparison. In addition, the contribution of each metabolite to the projection can be established based on their loadings, but more noteworthy, based on the directional loadings vectors as reflected in the bi-plots (Fig. 4 in the main text).

4.6 The vehicle / hippuric acid effect

The consumption of the flavoured water as vehicle is an intervention in its own right, given the relatively high concentration of sodium benzoate, used as a preservative. We have previously described the effect of the vehicle consumption[4]. From this investigation it became clear that hippuric acid dominates the urine profile, which we took in consideration when using flavoured water as vehicle for alcohol consumption — we applied a paired PLS–DA on the original data, excluding the information on hippuric acid, and compared the VIP values from this analysis to those from the unpaired PLS–DA which included hippuric acid. Both analyses produced 27 metabolites with a VIP > 1.0, with only the 12 metabolites defined as important indicators, and listed in Table 1, being common to both analyses when applying the additional criteria of WRT $p \leq 0.05$ and $|FC| \geq 1.5$. These results imply that the presence of hippuric acid in the data set did not influence the metabolites listed in Table 1, and, per implication, did not affect the outcome of the study.

4.7 Statistical software

Supplementary Table S3 lists the data analysis software used to perform the different statistical analyses discussed throughout this section.

Supplementary Table S3 List of statistical software

Analysis	Software
Univariate statistics	MATLAB and Statistics Toolbox Release 2012b, The MathWorks, Inc., Natick, MA, USA.
Multivariate statistics	PLS_Toolbox 8.2.1 (2016). Eigenvector Research, Inc., Manson, WA, USA 98831; software available at http://www.eigenvector.com.
Correlation analysis	T. Wei and V. Simko (2016). corrplot: Visualization of a Correlation Matrix. R package version 0.77. https://CRAN.R-project.org/ package=corrplot
Power curves	Dell Inc. (2016). Dell Statistica (data analysis software system), version 13. software.dell.com.
ML–PCA	Matlab m files created by Biosystems Data Analysis Group, Universiteit van Amsterdam. www.bdagroup.nl/content/Downloads/software. Copyright 2008
ASCA	Matlab using the statistics toolbox and code provided by

	Gooitzen Zwanenburg (available under APACHE Licence 2.0 http://www.apache.org/licenses/LICENSE-2.0.html).

5. Example of Informed Consent Form

INFORMED CONSENT TO PARTICIPATE IN EXPERIMENT WITH ETHICAL APPROVAL FROM THE NORTH-WESTUNIVERSITY

TITLE:

An investigation into the metabolic responses to acute alcohol consumption

AIM OF EXPERIMENT:

To determine the metabolic perturbations in young males in a fasted state, due to the consumption of a fixed acute dose of alcohol and/or NAD, as investigated by a metabolomics methodology.

INVESTIGATOR'S NAME: Cindy Irwin

SUPERVISOR: Prof. C.J. Reinecke

INVESTIGATOR SITE NAME & ADDRESS:

Centre for Human Metabonomics
NWU, Potchefstroom Campus
Private Bag X6001
POTCHEFSTROOM 2520
South Africa
Tel: 018 299 2309
Fax: 018 293 5248

INTRODUCTION

The North-West University's Centre for Human Metabolomics aims to investigate perturbations associated with human metabolism by means of a metabolomics approach. Metabolomics is a biochemical technique, involving a comprehensive study of low molecular weight biomolecules, commonly known as metabolites. Biochemical analysis of biological samples (such as urine or blood) provides a large comprehensive list of metabolites. The data are analysed by means of bioinformatics, a field of science incorporating statistical multivariate analysis, providing information used to determine/distinguish any potential irregularities within the metabolic profile. The focus is the identification of very specific metabolites that can statistically discriminate between normal and abnormal metabolic situations. These metabolites are known as biomarkers.

PURPOSE OF THE EXPERIMENT

This experiment constitutes part of the investigator's M.Sc. thesis involving the study into the metabolic perturbations associated with alcohol consumption. This experiment is aimed at determining the changes in the metabolite profile that occur after the consumption of an acute alcohol dose and what, if any, effect the administration of NAD together with the alcohol has to prevent these metabolic changes. To minimize variation, a homogeneous, defined experimental group, namely young males between the ages of 20 and 30 years in an overnight fasted state, will be used. The biological specimens used in this experiment will be a blood sample taken before the start of the experiment, as well as urine samples taken at defined intervals of time, followed by a metabolomics analysis.

PROTOCOL OF EXPERIMENT

Acute Alcohol and/or NAD Dose Study:

- Participants are required to abstain from alcohol consumption at least 48 hours preceding this experiment; as well as abstaining from consumption of food prior to initiation of the experiment (i.e. overnight fasted state).
- A questionnaire involving the basic clinical profile, including history of alcohol consumption, of the participant needs to be completed.
- An initial urine sample will be taken and labelled "0 hour" before alcohol and/or NAD dose is consumed.
- Group 1 participants will consume only the vehicle with which the alcohol will be mixed (i.e. lemon flavoured water) over a 15-minute period.
- Group 2 participants will consume a fixed dose of alcohol (Smirnoff vodka 43%, v/v, mixed with lemon flavoured water) of 1.5 g/kg body weight amount over a 15-minute period.
- Group 3 participants will consume the vehicle (i.e. lemon flavoured water), to which 50 mg of NAD has been added, over a 15-minute period.
- Group 4 participants will consume a fixed dose of alcohol (Smirnoff vodka 43%, v/v, mixed with lemon flavoured water) of 1.5 g/kg body weight, to which 50 mg of NAD has been added, over a 15-minute period.
- Urine and saliva for metabolomic investigations will be collected at an agreed time sequence over a period of 5 hours after alcohol and/or NAD dose first consumed, yielding 7 data points (consumption of water 15 minutes before a voiding is needed to facilitate obtaining urine samples).
- The experiment will be done in an environment compatible with clinical and ethical requirements
- A general practitioner will attend the early phases of the experiment (first 2 hours) and will be on call for the remaining part of the experiment.

NOTE: Urine samples must be labelled clearly and precisely and stored in a refrigerated environment and no additional alcohol should be consumed during the experimental time frame.

INFORMED CONSENT PROCEDURES

Participation in the project is fully voluntary. You are free to enquire about the experiment through the investigator and/or supervisor and, if agreeing to participate, the participant will be asked to sign this informed consent form. Should any participant request feedback on the outcomes of this study, such information can be made available to them.

It is required from all participants in this study to complete a questionnaire which provides information which is essential for the project. The participating physician will evaluate the information of all participants, and will approve their participation based on the information given in the questionnaire.

BENEFITS ASSOCIATED WITH THE STUDY

The Master's study, to which this experiment will contribute, is a research project aimed at better understanding the biological relationship between chronic/acute alcohol consumption and metabolic perturbations within humans. As in all cases, improved knowledge of the normal physiology will eventually yield to a better understanding and treatment of any deviation from normal physiology. The outcome of this research will be used by the researcher for a M.Sc. thesis and no reference will be included in the thesis regarding any individual who participated in the study.

PAYMENT OR REIMBURSEMENT

Participants will not be paid for their participation and do not contribute to the costs of the study. An amount of R100 will however be paid to each of the participants for travel expenses and other inconveniences that resulted from participation in the study as well as R50 for the light meal of choice after completion of the experiment.

CONFIDENTIALITY

All research records are confidential unless the law requires disclosure. No name or other personal identifying information of the participants will be used in any reports or publications resulting from this study. Data from this study will be used in an anonymous statistical analysis and reported as such by the NWU. No patient's identification details will be reported or made known to other parties.

VOLUNTARY PARTICIPATION AND CONDITIONS OF WITHDRAWAL

Your participation in this study is completely voluntary. You may choose not to participate in this study to which you are otherwise entitled.

CONSENT

I, ____, have read and understood the preceding information describing this research study and my questions have been answered to my satisfaction. I voluntarily consent to participate in this research study. I do not waive my legal rights by signing this consent form. I will receive a signed and dated copy of this consent form.

PARTICIPANT:

__________________________	______________________	________________
**Printed name**	**Signature**	**Date**

INVESTIGATOR:

________________________	_____________________	_______________
**Printed name**	**Signature**	**Date**

PHYSICIAN:

________________________	_____________________	_______________
**Printed name**	**Signature**	**Date**

6. References

1. Cohen, J. *Statistical Power Analysis For Behavioural Sciences.* Second edition. (Hillsdale, NJ: Erlbaum, 1988).
2. Imam, A., Mohammed, U. & Abanyam, C. M. On consistency and limitation of paired t-test, sign and Wilcoxon sign rank test. *IOSR Journal of Mathematics* **10(1)**, 1–6 (2014).
3. Villez, K., Steppe, K. & De Pauw, D. J. W. Use of unfold PCA for on-line plant stress monitoring and sensor failure detection. *Biosystems Engineering* **103**, 23–24 (2009).
4. Irwin, C. et al. Contribution towards a metabolite profile of the detoxification of benzoic acid through glycine conjugation: An Intervention Study. PLOS ONE 11(12), e0167309. doi:10.1371/journal. pone.0167309 (2016).
